# Nanoscale chemical imaging of phagocytosis: A battle for metals between host and microbe

**DOI:** 10.1016/j.jbc.2025.110485

**Published:** 2025-07-16

**Authors:** Nadeem Ullah, Björn De Samber, Nathalie Uwamahoro, Stijn J.M. Van Malderen, Linda Sandblad, Sylvain Bohic, Peter Cloetens, Laszlo Vincze, Constantin F. Urban

**Affiliations:** 1 Department of Clinical Microbiology, Umeå University, Umeå, Sweden; 2 Umeå Centre for Microbial Research (UCMR), Umeå University, Umeå, Sweden; 3 Department of Analytical Chemistry, Ghent University, Ghent, Belgium; 4 Department of Chemistry, Umeå University, Umeå, Sweden; 5 Umeå Centre for Electron Microscopy, Umeå University, Umeå, Sweden; 6 European Synchrotron Radiation Facility, Grenoble, France

**Keywords:** neutrophils, PMNs, immunology, infection, synchrotron, X-ray fluorescence, nanoprobe, X-ray nanohomotomography, *S. cerevisiae*, metallomics, nutritional immunity

## Abstract

The human body employs nutritional immunity to restrict essential micronutrients, such as zinc, from invading pathogens, impeding their growth and replication. Here, we applied an advanced nanochemical imaging technique, synchrotron radiation-based X-ray fluorescence (SR-XRF), on vitrified polymorphonuclear neutrophils (PMNs) during the occurrence of phagocytosis of *Saccharomyces cerevisiae*. Nanoscopic SR-XRF provided trace elemental distributions at 50 nm spatial resolution, revealing the metal interplay between PMNs and *S. cerevisiae*. Our results were complemented with X-ray holographic nanotomography (XNH), confirming phagocytosis and providing complementary intracellular morphological information. A systematic decrease in zinc was observed between free and phagocytosed *S. cerevisiae* within the same XRF maps, suggesting active zinc depletion by PMNs. Other elements, such as sulfur, show an increase in the phagosome, likely indicative of the increase in proteins in the vicinity of phagocytic events. Through 2D/3D nanoimaging and time-lapse microscopy, we confirmed the reduction of zinc within phagocytosed yeast. Hence, our findings challenge the currently accepted hypothetical model that PMNs intoxicate engulfed microbes with an overwhelming influx of zinc ions into the phagosome. Furthermore, antimicrobial assays demonstrated that *S. cerevisiae* can cope well with sudden zinc spikes. Even high zinc concentrations imposed on *S. cerevisiae* grown under zinc-limiting conditions did not have adverse effects on viability. Contrarily, *S. cerevisiae* was more resistant to phagocytic killing by PMNs when grown under high zinc concentrations before infection. Our findings further consolidate zinc deprivation as an effective antimicrobial strategy. A better understanding of the metal deprivation mechanisms could inspire new exploitable targets for antimicrobial therapies.

Phagocytic cells play a central role as part of the innate immune system, in which they serve as the first line of defense against invading microorganisms (1, 2, 3). One of the cells with the capacity for engulfment is the polymorphonuclear neutrophil (PMN). Of note, the three-letter abbreviation and the shortened term ”neutrophil” are used interchangeably throughout the text. Neutrophils are terminally differentiated, non-proliferative cells armed with a large antimicrobial arsenal (2, 4, 5). They recognize and track microbial invaders using chemotactic migration along concentration gradients of chemoattractant molecules, leading them to the infection site (6, 7). Upon contact with pathogens, PMNs use phagocytosis to engulf and eliminate microorganisms (6). Phagocytosis is characterized by membrane extensions that eventually surround the pathogen. A membrane vesicle (phagosome) with the pathogen inside is formed and engulfed. Then, neutrophil granules present in the cytoplasm fuse with the phagosome, giving rise to a so-called phagolysosome. Upon fusion, the antimicrobial content of the granules, comprised of antimicrobial peptides, enzymes, and metal-binding proteins, is released into the phagolysosome to eradicate the pathogen (8, 9). In addition, the NADPH oxidase complex is assembled at the phagolysosomal membrane, releasing superoxide and other reactive oxygen species (ROS) into the microbe-containing vesicle. Oxidative and antimicrobial components are combined in the phagolysosome to aid destruction of microbes (10).

By exposing microbes to metal-binding proteins, neutrophils can modulate the availability of micronutrients *via* a process termed nutritional immunity. The deprivation of micronutrients, mostly metal ions, by immune cells is used as a successful strategy to combat infections, since those metal ions are crucial for the survival of every living organism (11). Hence, restricted access to these metal ions inhibits microbial growth. To be able to survive in such host milieus, bacteria and fungi have, among others, developed iron (Fe) acquisition systems to overcome the host-restricted access to this essential metal. Similar sequestration mechanisms have also been reported for other metal ions, including zinc (Zn), copper (Cu), and manganese (Mn) (12, 13, 14). Metal nutrients are known to play a key role during infection (15), and as a logical result, a battle for those nutrients arises during infection between host immune cells and pathogens (15). Moreover, there is also a growing body of evidence that metal chelating proteins, such as for instance S100A8/A9, are deployed to deprive pathogens of essential metals (16). S100A8/A9 makes up to 40% of the PMN cytoplasm and chelates essential metals such as manganese and zinc, resulting in its high antimicrobial properties (12, 17, 18). Of note, besides depriving microbes of essential metals, the host can also accumulate high quantities of metal ions to generate toxic environments for microbes. Space-limited and confined environments, for instance, the phagolysosome, are ideal niches for such intoxication attempts. Macrophages, another type of professional phagocyte, have been shown to increase copper ion concentrations in their phagolysosomes to such levels that microbes succumb to the copper intoxication (19). Hence, determining under which circumstances immune cells either deprive microbes of metals or accumulate toxic concentrations of metals is important to better understand innate immune responses.

Currently, only a few analytical methods are capable of visualizing trace-level metal distributions at the subcellular level (19). Synchrotron radiation-based nanoscopic X-ray fluorescence imaging (XFI) is an analytical methodology of this kind providing multi-element maps at trace level and subcellular spatial resolution (20). Lately, a limited number of hard X-ray nanoprobes at third-generation synchrotron sources have been equipped with cryogenic sample environments (21, 22), enabling the analysis of frozen-hydrated cells in a close to native state. A previous nanoscale XFI report described the elemental content within the mycobacterial phagosome of macrophages infected with *Mycobacterium smegmatis*. The study showed that the iron concentration in phagosomes decreased over time but increased in other areas within the macrophages, which were infected with pathogenic mycobacteria (23).

Phagocytic activity from neutrophils has been extensively studied, and the use of the yeast *Saccharomyces cerevisiae* comprises a good model for this purpose (24). Compared to bacteria, yeast cells provide several advantages for their use in XFI studies: Yeast cells are larger than bacterial counterparts, hold higher absolute amounts of trace elements than prokaryotes, and are phagocytosed in comparable rates by human neutrophils (25, 26). Since the elemental distributions were obtained from vitrified specimens, the phagocytic process and flow of elements between phagocyte and microbe were undisturbed and not potentially altered by chemical fixation.

To date, it remains uncertain whether neutrophils can remove zinc ions from microbes within the phagosome or whether neutrophils shuttle zinc ions into the phagosome to surmount tolerable levels. Herein, we attempt to better understand which strategy PMNs use regarding the trace element zinc during phagocytosis of microbes. We performed nanochemical imaging of cryofrozen PMNs phagocytosing *S. cerevisiae* at different time points to obtain data on the distribution of zinc concentrations at the subcellular level. X-ray holographic nanotomography (XNH) was used to additionally provide detailed morphological information of these phagocytic events intracellularly. Furthermore, we demonstrate that neutrophil phagocytic killing of yeast was increased in low zinc and reduced in high zinc environments.

## Results

### Quantitative nanochemical imaging of opsonized *S. cerevisiae* engulfed by neutrophils

**Figure 1:**
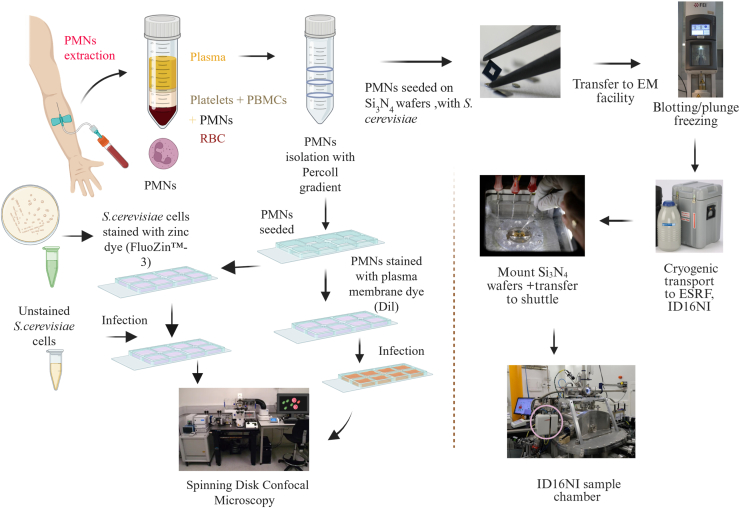
**Overview of procedures used.** Illustrated are neutrophil extraction for spinning disk confocal microscopy (*left*) and cryogenic workflow (*right*) for nanochemical imaging at beamline ID16NI (ESRF). *Left:* PMNs were membrane-stained and seeded into 8-well microscopy slides. Subsequently, opsonized *S. cerevisiae* cells, either labeled with a fluorescent zinc probe or unlabeled as control, were added to the wells. The samples were analyzed using a spinning disk confocal microscope and the zinc content in *S. cerevisiae* cells was quantified *via* image analysis as described in Experimental Procedures. *Right*: Silicon nitrate (Si_3_NO_4_) wafers were placed into the wells of microplates in which PMNs were seeded in cell culture medium. Opsonized *S. cerevisiae* was added, and the plates were incubated under cell culture conditions. After different incubation times, the wafers were removed and briefly rinsed with deionized water to remove residual media contents. The wafers were mounted on a plunge freezing equipment and instantly vitrified using liquid ethane. Subsequently, the wafers were placed in pre-labeled containers and stored in liquid nitrogen until use.

*S. cerevisiae* was used as a model microbe for infection studies with PMNs since, at the time of the experiment, biosafety regulations did not allow pathogens within the high vacuum of the beamline setup of the ID16NI nanoprobe (Fig. 1). Wafers used for sample analysis at ID16 NI were placed at the bottom of microplate wells. PMNs were seeded into these wells, and *S. cerevisiae* cells were added at different time points. The interacting immune cells and microbes attached to the wafers, which subsequently were removed from the microplates and directly plunge-frozen using liquid ethane. Instant cryopreservation of the samples ensured that their elemental composition was not altered, which might occur during chemical fixation. Once inserted into the X-ray nanoprobe, candidate phagocytic events were selected based on optical microscopy of the cryo-frozen wafers (Fig. 1). Then, so-called “coarse” XRF scans were performed with lower spatial resolution (200 nm step size, approximately 10 min per scan) to confirm whether *S. cerevisiae* cells were in close interaction with PMNs. Up to eight phagocytic events on the same silicon nitride wafer were identified in this manner, after which high-resolution XRF scans were taken (50 nm step size, approx. 1h per scan).

**Figure 2:**
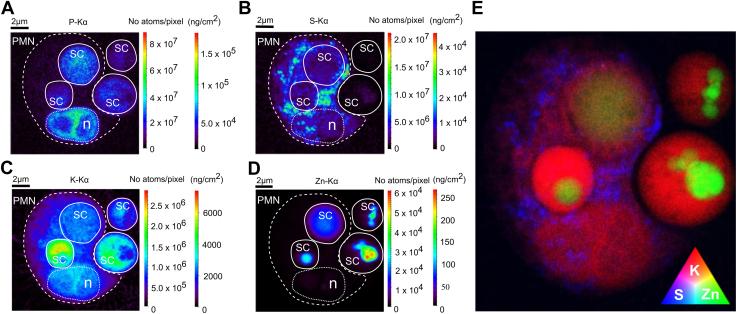
**Nano-XRF elemental heat maps show concentrations of elements in PMNs and *S*. *cerevisiae*.** Elemental distribution maps, *left*: quantified elemental distribution (first color bar in no. atoms/pixel, and second in ng/cm^2^) of P (*A*), S (*B*), K (*C*), and Zn (*D*) within a single, vitrified PMN (*circled in dashed line*) in the process of phagocytosing opsonized *S. cerevisiae* (*circled with continuous line*). The neutrophil nucleus is indicated with 'n' (*circled with a fine-striped line*). PMNs were incubated with *S. cerevisiae* for 3 h. A clear decrease in the areal concentration of Zn can be noticed comparing unengulfed and engulfed *S. cerevisiae*. (*E*) RGB representation of the elements K (*red*), Zn (*green*) and S (*blue*); scaling is optimized per element. Pixel size is 50 nm, dwell time was 50 ms. Data was acquired at the ID16NI 'nano-imaging' beamline at the ESRF in Grenoble, France.

To illustrate the elemental landscape of the interaction of yeast cells and PMNs, a single SR-XRF scan at high resolution (50 nm step size) is shown in (Fig. 2). The scan depicts a PMN with two fully engulfed yeast cells, one yeast cell just prior to phagocytosis and another yeast cell in close proximity but outside the PMN. Quantitative distributions of the elements phosphorus (P), sulfur (S), potassium (K), and zinc (Zn) are shown in (Fig. 2, *A*–*D*). For enhanced illustration, we included an RGB (red, green, blue) representation of elements potassium, zinc, and sulfur (Fig. 2*E*). For this representation, scaling was adjusted for optimal visibility. The location of the cell membrane of the PMN was marked with a dashed white line, while *S. cerevisiae* was marked with an uninterrupted line. In the phosphorus map, next to the PMN, a larger and a smaller *S*. *cerevisiae* cell are visible which appear to not yet have separated. Most probably, the smaller yeast represents a bud of the larger cell. Within the PMN, two already engulfed and apparently unseparated *S*. *cerevisiae* cells can be observed, again one being larger than the other. As outlined, the nano-XRF elemental maps provide structural information of different time points of phagocytic events in the absence of light microscopic information and confirm that the method can be used to study phagocytosis and underlying elemental fluxes. The structure at the bottom of the PMN, indicated with 'n' and circled with thinner dashed lines, represents a part of the lobulated neutrophil nucleus. In the sulfur heat map, we observed granular, sulfur-rich accumulations in the cytoplasm of the PMN, which could be indicative of the presence of protein-rich neutrophil granules. The granular vesicles contain the bulk of antimicrobial proteins present in PMNs (27, 28). From all elemental maps, the potassium (K) distribution generally provided the most morphological features of the neutrophil and showed relatively high concentrations in *S. cerevisiae*, both engulfed and unengulfed (the latter labeled as 'free' *S. cerevisiae*). Within the heat map, limit of detection (LOD) of potassium was determined to be 28 ppm or 730 μM (Figs. S1–S3 contain more information on the determination of LODs). Interestingly, the areal concentrations of zinc (LOD 0.44 ppm or 6.7 μM) were highest for free *S. cerevisiae* outside the PMN, reaching up to 250 ng/cm^2^. A clear structural clustering of zinc is visible in the free *S. cerevisiae,* probably reflecting subcellular storage of zinc within the vacuole. Contrarily, the subcellular pattern appeared to be lost in the engulfed yeast cell. More importantly, we observed a clear decrease in mean areal concentration of zinc for the following series: 'free *S. cerevisiae*' > 'phagocytosed *S. cerevisiae*' > 'entire PMN' > 'PMN – phagosome'. Overall, visual investigation of elemental maps provided structural information and allowed the investigation of phagocytic events. The elemental maps suggested redistribution and reduction of Zn within engulfed *S. cerevisiae* cells. In what follows, differences in zinc concentrations were statistically tested across all analyzed XRF maps and cluster analyses of different subcellular areas were performed.

### Combined nanochemical and morphological imaging of opsonized *S. cerevisiae* engulfed by neutrophils

**Figure 3:**
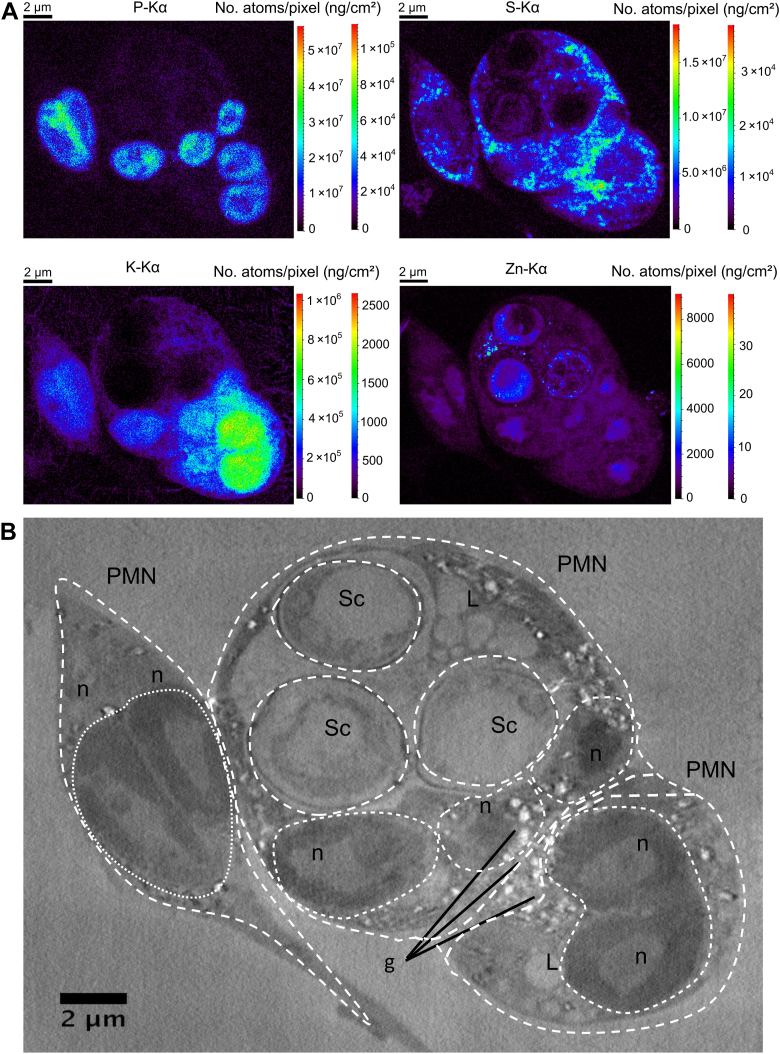
**Yeast cells engulfed by PMNs appear to be element exhausted.** (*A)* Quantified elemental distribution (first bar in no. atoms/pixel, and second bar in ng/cm^2^) of P, S, K, and Zn of specimens of vitrified PMNs (*circled in dashed line*) with engulfed *S. cerevisiae* (Sc) inside (One of three PMNs in the center of the image). PMNs were exposed to *S. cerevisiae* for 3 h. Areal concentration maps were calculated using a dedicated quantitative method, including normalization, ice thickness determination, ice self-absorption correction and background subtraction. Ice thickness of the sample was estimated to be 9.4 μm using the K-K_α_K_β_ ratio. (*B*) Virtual section of the same sample obtained *via* XNH (Sc and PMN, *circled in dashed line*), 'L' indicates the possible formation of a phagolysosome, 'g' indicates antimicrobial granules. See Movie S1 for a more comprehensive additional visualization of the XNH procedure.

Nanoscale element imaging of three PMNs of which only one immune cell in the center has engulfed 3 *S. cerevisiae* cells demonstrates that internalized yeast cells appear to be virtually emptied out of elements, such as phosphorus, potassium, and sulfur (Fig. 3*A*). Some dotted patterns of low levels of zinc remained within the engulfed yeast cells. However, as before (Fig. 2), the vacuolar storage compartment organization appears to be lost upon internalization. To gather more structural information, we conducted morphological imaging of the same specimen using XNH, of which a single reconstructed slice is provided here (Fig. 3*B*). The entire sequence of reconstructed slices can be found as a digital TIFF file (Movie S1). The denser perinuclear region of the PMN nucleus ('n') is visible as a darker region. Bright (*i.e.*, lower density) granules ('g') are visible in the cytoplasm of the PMN, corresponding to the sulfur-rich regions in the nano-XRF map. 'L' indicates a region in which granules might be accumulating for fusion with the phagosome to form a phagolysosome (Fig. 3*B*). Although not fully reaching the resolution obtained with cryo-electron microscopy (29, 30), our measurements showcase that XNH imaging allows the investigation of relatively thick vitrified cellular specimens. Samples embedded in ice layers with diameters of a few tens of micrometers are typically considered thick. Moreover, the method used is essentially non-destructive and enables correlation of the obtained elemental distributions with a wide diversity of cellular organelles (nuclei, antimicrobial granules, phagosomes, *etc.*). As a reference, we used elemental maps derived from separate specimens of monocultures of *S. cerevisiae* and of neutrophils Figs. S4, *A*, *B* and S5, *A*, *B*). The presented XNH reconstruction clearly demonstrates that elemental maps, which are 2D projections, can be correlated with phagocytosing PMNs with engulfed yeast cells inside.

As indicated earlier, zinc levels within yeast cells seemed to be reduced upon phagocytosis by neutrophils (Figs. 2 and 3). For other elemental distributions, such as potassium, we observed a similar trend. The levels of this element appeared to be higher in free *versus* engulfed *S. cerevisiae*. For sulfur, however, we observed seemingly higher concentrations in engulfed *versus* free *S. cerevisiae*, probably caused by the phagolysosomal release of sulfur-rich granule content.

### Neutrophils actively deprive zinc from phagocytosed *S. cerevisiae*

**Figure 4:**
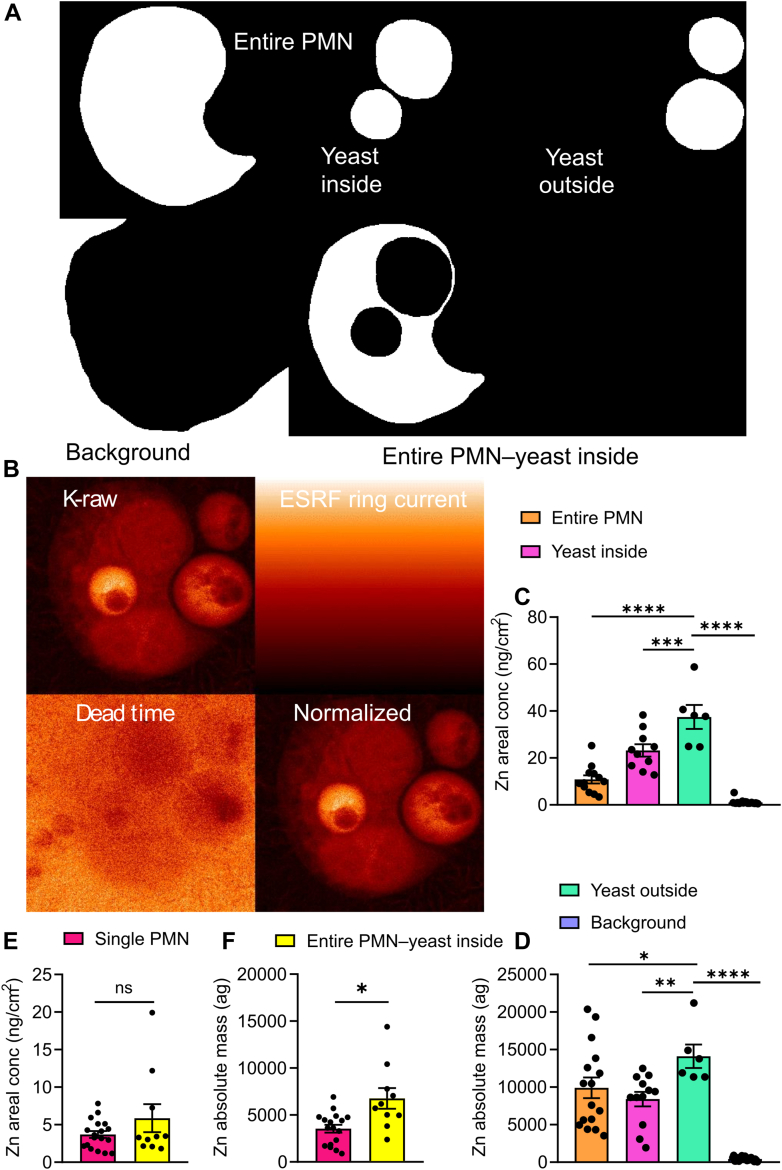
**Zinc quantification and normalization of the nano-XRF heat maps.** (*A* and *B)* representative binary map, cluster calculations and normalization strategy for the different areas within analyzed XRF maps depicting entire PMN (PMNs containing internalized *S. cerevisiae*), yeast inside (engulfed yeast cells), yeast outside (unengulfed *S. cerevisiae*), single PMN (phagocyte in the absence of *S. cerevisiae*) and background. (*C* and *D*) Background-corrected mean areal Zn concentrations (expressed in ng/cm^2^) and absolute mass (in ag) of nano-XRF maps. Zn concentrations were calculated for 5 different cluster areas as described in the text: 1) entire PMN, 2) yeast inside, 3) yeast outside, and (*E* and *F*) 4) entire PMN – yeast inside 5) single PMN. Results were shown as the mean ± SEM. *p-*values were calculated using an ordinary one-way ANOVA with *post hoc* Tukey's and Dunnett's test adjustment for multiple comparisons. The number of independent samples used is indicated by the dots accompanying each bar within the displayed graphs. *p-*value ∗*p <* 0.01, ∗∗*p <* 0.001, ∗∗∗*p <* 0.0001 were considered statistically significant, while 'ns' indicates not significant.

In order to get a deeper understanding of the metal dynamics during phagocytosis, with a special focus on zinc, we calculated mean (background-corrected), areal concentrations, and absolute mass. We performed these analyses for control samples of PMNs alone and of yeast cells alone, and for samples of PMNs infected with *S. cerevisiae*. Specific cluster areas were quantified as well: 1) Area of a PMN, including any phagocytosed *S. cerevisiae* (‘entire PMN’). 2) Area of *S. cerevisiae* yeast cells internalized by a PMN (‘yeast inside’). 3) Area of a PMN from which the area of the internalized *S. cerevisiae* cells was subtracted (‘entire PMN – yeast inside’). 4) *S. cerevisiae* located outside of PMNs (‘yeast outside’). 5) Area of a PMN containing no *S. cerevisiae* (‘single PMN’) (Fig. 4, *A* and *B*). Mean elemental zinc concentrations and absolute mass of the samples across 2 different beamtime experiments were quantified. Different areas for PMNs and yeast cells were defined (refer to the Experimental Procedures section for more details of the cluster calculations and normalization strategy). Overall mean zinc content for a total of analyzed PMNs was found to be 10.81 ng/cm^2^. Also, for 3 *S. cerevisiae* in the absence of PMNs, mean areal concentrations of zinc were determined as 26.30 ng/cm^2^, confirming the much higher zinc concentration of *S. cerevisiae* compared to PMNs. Thus, the previously assumed antimicrobial strategy to increase zinc concentrations within the phagosome to toxic levels for engulfed microbes seemed less likely to be applicable for neutrophils.

In the next step, all nano-XRF maps containing examples of 'yeast outside' and 'yeast inside' within the same XRF map were quantitatively analyzed (Fig. 4, *C* and *D*). In this comparison, available samples ranged from n = 6 for ‘yeast outside’ to n = 16 for ‘entire PMN’. Mean zinc concentrations calculated from the different nano-XRF maps varied significantly within every cluster type (*i.e.*, ‘entire PMN’, ‘yeast inside’, ‘yeast outside’). However, mean areal zinc content and absolute mass of the phagocytosed *S. cerevisiae* (‘yeast inside’) were consistently lower compared to the unengulfed *S. cerevisiae* (‘yeast outside’), which suggests a net zinc flux from *S. cerevisiae* to PMNs. In agreement, the mean absolute mass of zinc in samples of ‘entire PMNs – yeast inside’ was significantly higher than the absolute mass of zinc in samples of PMNs with no *S. cerevisiae* inside (‘single PMNs’). The zinc area concentration was somewhat higher in phagocytosing PMNs as well. However, the statistical test lacked significance, probably because the extracted zinc is quickly diffused throughout the PMN (Fig. 4, *E* and *F*).

### Zinc depletion of *S. cerevisiae* upon interaction with neutrophils

**Figure 5:**
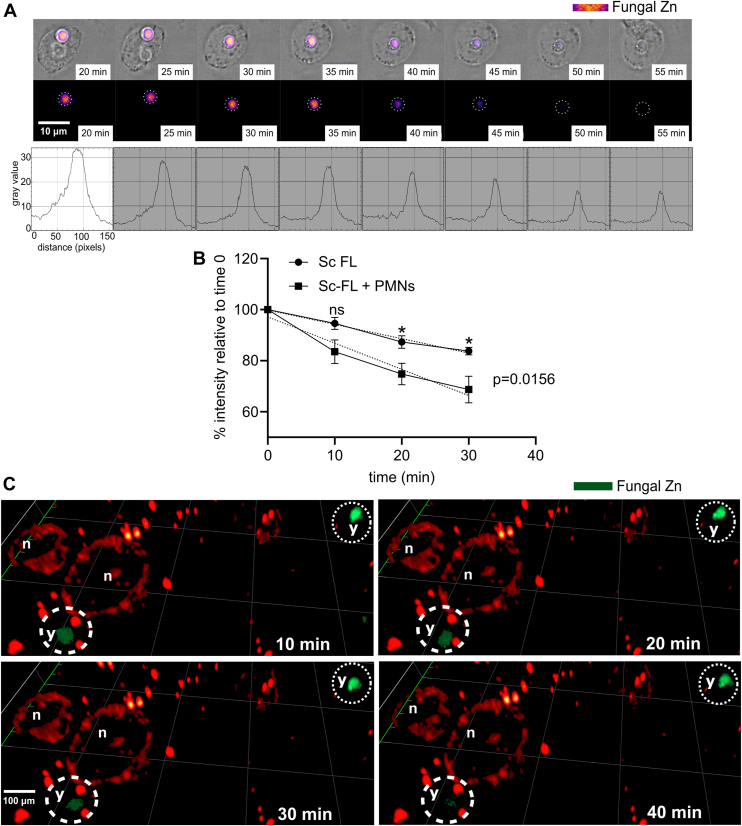
**Fungal zinc depleted from engulfed and adjacent yeast cells in a PMN-dependent manner.** (*A)* stills of time-lapse fluorescent microscopy at indicated time points showing decrease of intracellular Zn levels by FluoZin-3-AM staining an engulfed *S. cerevisiae.* As complementation, plotted profiles of *gray* values (pixels) of the FluoZin-3-AM intensity over time are also shown. The plots were generated from fluorescent images and the areas inside the *dotted circles*. Representative microscopic images demonstrate Zn depletion from engulfed yeast. (*B*) Percentage fluorescence intensity of *S. cerevisiae* cells in the absence (Sc FL) and presence of PMNs (Sc FL + PMNs). (*C*) Representative 3D images acquired using spinning disk confocal microscopy of *S. cerevisiae* stained for Zn content with FluoZin-3-AM (*green*) and PMNs (stained with DiI, *red*). An *S. cerevisiae* cell is shown in close vicinity to a PMN on the verge of being phagocytosed displaying depletion of fungal zinc over time (encircled with *white dotted line*) while the fungal Zn remains virtually stable in a distant cell (encircled with a *white dashed line*). Quantification in (*B*) stems from n = 3 (3); three biological replicates with technical triplicates each. Significant differences between the slopes (*p* = 0.0156) were determined through linear regression and an unpaired *t* test was used to compare individual time points. *p*-values indicate statistical significance at ∗*p* < 0.01, while ‘ns’ equals not significant. Scale bars represent 10 μm.

To further investigate zinc distribution during the interaction of PMNs and *S. cerevisiae*, we decided to use microscopical methods (Fig. 1). In doing so, we took advantage of fluorescent probes that indicate the content of free zinc. To monitor zinc depletion from *S. cerevisiae*, yeast cells were labeled with FluoZin-3-AM (green), while PMNs remained unstained. The decrease in zinc levels of engulfed *S. cerevisiae* was visualized using time-lapse fluorescent microscopy, represented as stills from indicated time points (Fig. 5*A*, upper panel). Zinc staining is depicted as the fluorescence intensity of stained *S. cerevisiae,* ranging from yellow (high) to purple (low). Histogram plots of pixel values support the notion of zinc decrease in engulfed *S. cerevisiae* in a more quantitative manner. Hence, zinc appears to decrease within engulfed yeast cells, while zinc levels remain fairly stable in control samples lacking PMNs. The slight reduction of fluorescence in control samples probably stems from mild bleaching effects (Fig. 5*B*).

Additionally, the samples of PMNs infected with FluoZin-stained *S. cerevisiae* were analyzed using spinning disk confocal microscopy and 3D rendition of live cell imaging every 10 min for 40 min at several positions. To facilitate visualization of PMNs during fluorescence microscopy, PMN membranes were stained with dioctadecyl-3,3,3′,3′-tetramethylindocarbocyanine perchlorate (DiI). Upon phagocytosis of yeast cells by PMNs, the zinc level in yeast cells (dotted line) clearly decreased (Fig. 5*A*, upper panels). In contrast, the zinc staining of a distant yeast cell, which was not interacting with a phagocyte, remained at similar levels throughout the experiment.

Taken together, these microscopic analyses demonstrate that *S. cerevisiae* is deprived of intracellular zinc levels in the presence of PMNs, rendering the notion that PMNs accumulate zinc to intoxicate phagocytosed microbes highly unlikely.

### High zinc pulses have no antimicrobial effect on *S. cerevisiae* as determined by multiple independent assays

Next, we aimed to demonstrate the effect of a sudden and vigorous increase in zinc concentration on *S. cerevisiae*. For this purpose, we cultivated *S. cerevisiae* in a low zinc medium (LZM, virtually no zinc present), in a mid-range zinc medium (MZM = 2.5 μM zinc concentration) and in high zinc medium (HZM = 25 μM zinc concentration). All other media components remained the same between these culture media as specified in Experimental Procedures. After incubation to an optical density (600 nm) of 1.5 to 3, we divided the cultures in two parts. One part was subjected to a sudden addition of zinc sulphate (zinc pulse) to a final concentration of 500 μM, while to the other part was mock-treated with the relevant growth medium (0 μM, 2.5 μM or 25 μM zinc), respectively.

**Figure 6:**
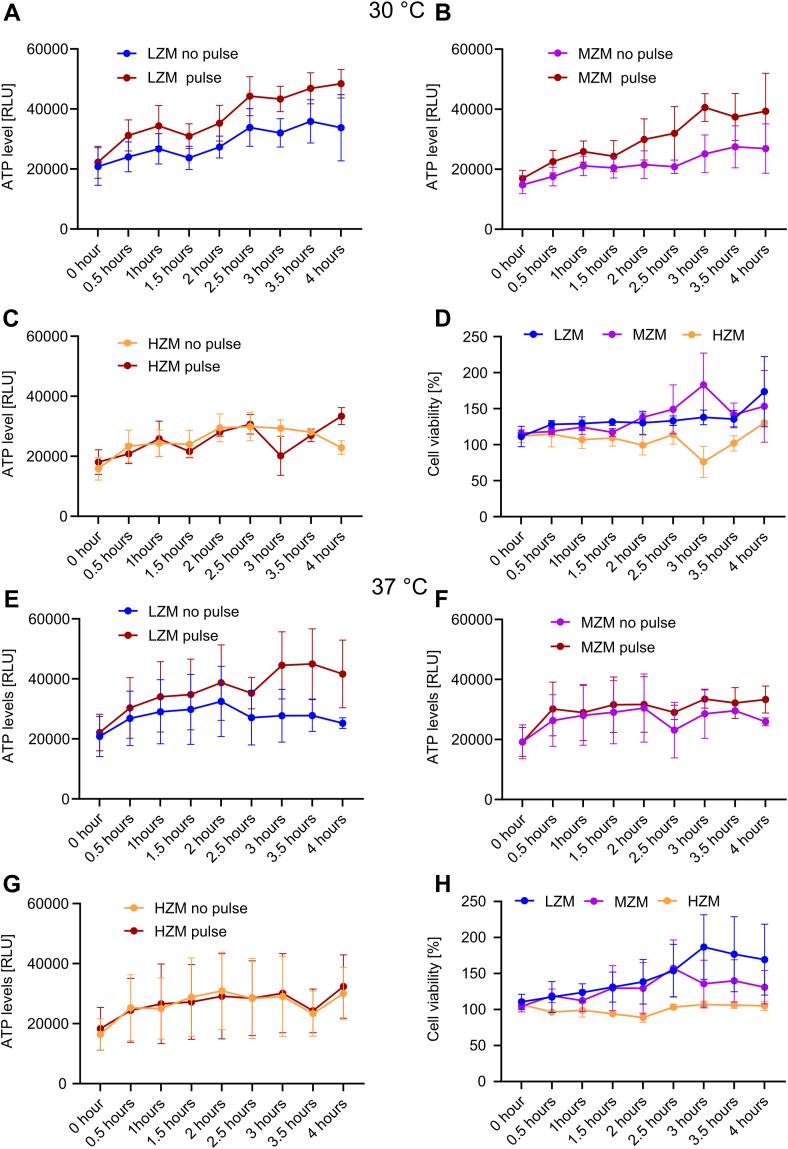
**ATP quantification to determine viability of *S. cerevisiae* upon exposure to zinc pulses.***Panels* (*A*–*C* and *E*–*G*) present ATP levels of *S. cerevisiae* grown either at 30 °C (*A*–*D*) or 37 °C (*E*–*H*) with or without Zn pulse. ATP was quantified at different time points between 0 and 4 h after the pulse. *S. cerevisiae* was grown in LZM (0 μM Zn), MZM (2.5 μM Zn), and HZM (25 μM Zn), with addition of 500 μM ZnSO_4_ (Zn pulse) or mock treatment with addition of medium only (no pulse). *Panels* (*D* and *H*) present percental ratio of cell viability of the *S. cerevisiae* samples exposed to the pulse compared to the mock-treated samples. The data were shown as the mean ± SEM n = 3(3), where “n” represents the number of biological replicates with the number of technical replicates within individual experiments in brackets. Statistical analysis was performed using a one-way ANOVA test for multiple comparisons or a non-parametric *t* test with Welch's correction showing no statistically significant effect of the pulse on ATP levels.

First, we analyzed the viability of *S. cerevisiae* by quantification of ATP using a luciferase assay. Cellular ATP levels of *S. cerevisiae* were determined at different time points ranging from 0 h to 4 h after exposure to a 500 μM zinc or mock pulse (Fig. 6, *A*–*C*). The assays were conducted at 30 °C and 37 °C, the latter representing body temperature. *S. cerevisiae* grown in LZM as well as in MZM showed increased growth upon zinc pulse as compared to the control medium (Fig. 6, *A* and *B*). *S. cerevisiae* grown in HZM was virtually unaffected by the zinc pulse (Fig. 6*C*). We recorded a slight dip in viability after 3 h of incubation in NZM with zinc pulse, probably derived from one outlier measurement. To corroborate, we calculated the percentage survival of LZM, MZM, and HZM with the respective Zn pulse condition (Fig. 6*D*). In each pair of conditions, percentage survival exceeded or ranged close to 100 percent, confirming that the zinc pulse had no killing or inhibiting effect on *S. cerevisiae*. Similar results were obtained at 37 °C, demonstrating that sudden increases in zinc concentration also have no adverse effects on *S. cerevisiae* at body temperature (Fig. 6, *E*–*H*).

**Figure 7:**
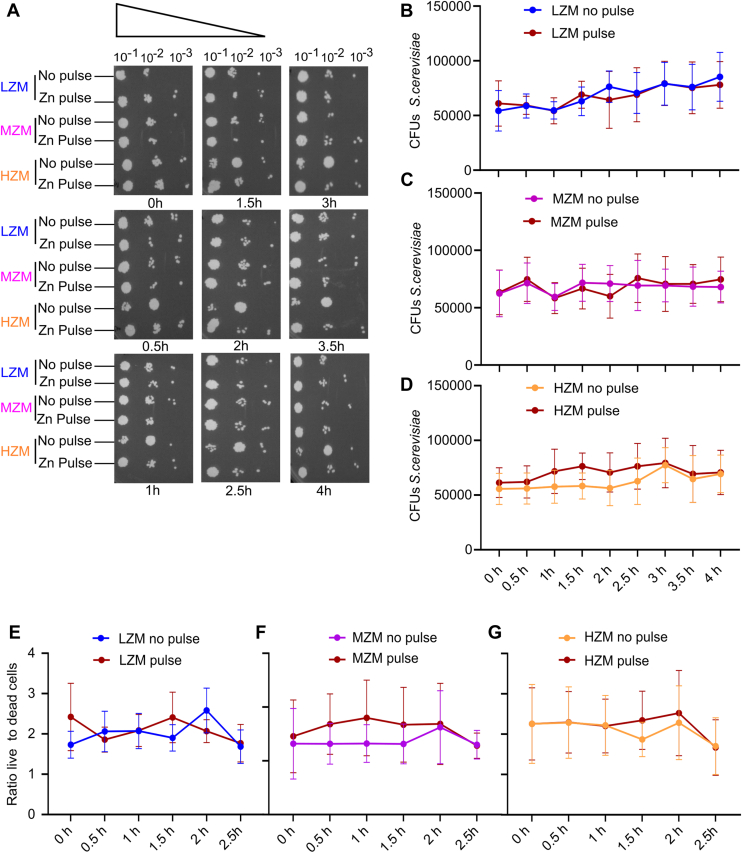
**Zinc pulses do not adversely affect viability of *S. cerevisiae*.** (*A*) representative images of serially diluted 10-fold and plated *S. cerevisiae* from different preculture conditions with or without Zn pulse. (*B–D*) CFU enumeration of *S. cerevisiae* grown in LZM (0 μM Zn), MZM (2.5 μM Zn), and HZM (25 μM Zn), with addition of 500 μM ZnSO_4_ (Zn pulse) or mock treatment with addition of medium only (no pulse). (*E*–*G*) quantification of live and dead yeast cells after the Zn pulse or mock pulse at different time points ranging from 0 to 2.5 h. The ratios of live to dead yeast cells were calculated. The data is shown as the mean ± SEM n = 3 (3), where “n” represents the number of biological replicates with the number of technical replicates within individual experiments in brackets. *p*-values were calculated using a two-way ANOVA test or a non-parametric *t* test with Welch's correction which showed no significant negative effect of Zn pulse on *S. cerevisiae* viability.

Secondly, we used a spot test as an alternative method for the assessment of *S. cerevisiae* viability exposed to a zinc pulse. After LZM, MZM, and HZM cultivation at 30 °C in the presence and absence of a zinc pulse, similarly as described above, *S. cerevisiae* was serially diluted on synthetic complete (SC) media agar plates. Colony-forming units (CFUs) on plates were photographed (Fig. 7*A*) and CFUs were counted at suitable dilution steps (Fig. 7, *B*–*D*). CFU remained largely unaltered when comparing the relevant cultivation pairs LZM and MZM ± zinc pulse (Fig. 7, *B* and *C*). A slight difference between HZM cultivation with zinc and mock pulse was recorded, yet again, the viability in the presence of the zinc pulse was slightly higher (Fig. 7*D*). Overall, no inhibitory or killing effect of the zinc pulse could be observed. Minor differences in ATP and spot test results might stem from increased cellular activity resulting from the addition of zinc in *S. cerevisiae* previously grown under low zinc conditions, which was not reflected by the overall CFU count. Notably, the percentage of *S. cerevisiae* CFUs with zinc pulse compared to CFUs with mock pulse was positively increased, particularly when comparing *S. cerevisiae* grown with LZM to *S. cerevisiae* grown with MZM or HZM (Fig. S6), corroborating the notion that a zinc pulse does not have any adverse effects on *S. cerevisiae*.

Finally, we applied fluorescent live and dead staining on *S. cerevisiae* cells, comparing zinc-pulsed and mock-pulsed yeast cells. With a similar setup as described above, the fluorescent emission of the dyes was recorded and subsequently quantified using a cell imaging multimode reader. pre-grown *S. cerevisiae* cultures were evenly seeded into wells of 96-well plates and either treated with a zinc pulse or mock pulse. Upon addition of the live and dead staining, cells were incubated at 37 °C and analyzed for 2.5 h. The ratios of live to dead cells remained fairly unchanged, comparing zinc-pulsed cells to mock-pulsed cells (Fig. 7, *E*–*G*). No adverse effects of the zinc pulse could be observed, irrespective of the previous growth conditions (LZM, MZM, and HZM). The relatively high percentage of dead cells in this assay is probably owed to the seeding before analysis and the addition of the fluorescent stains to the samples. Notably, the number of dead cells is independent of the presence of a zinc pulse (Fig. S7, *A*–*C*).

In summary, these independent microbial assays clearly demonstrate that *S. cerevisiae* is considerably resilient to zinc intoxication. The concentration of 500 μM is approximately 30 to 54 times higher than the median concentration of zinc in human plasma (31, 32, 33). That PMNs could gather enough zinc to surmount microbial tolerability against zinc intoxication seems thus rather unlikely. In addition, zinc tolerability of *S. cerevisiae* is neither dependent on the zinc concentration present in the growth medium nor on temperature differences.

### Low zinc levels in yeast cells increase the susceptibility to phagocytic killing by neutrophils

**Figure 8:**
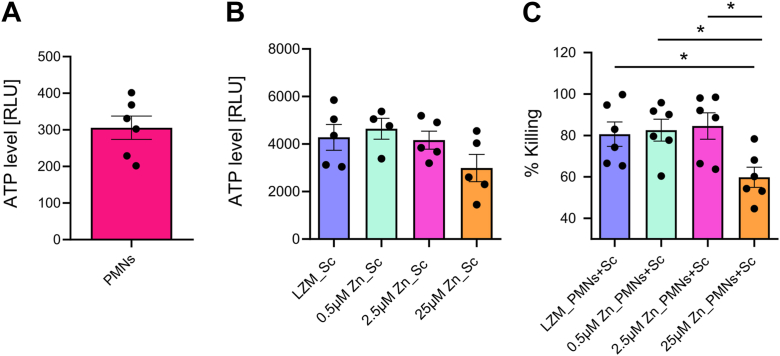
**Low zinc level in yeast enhances phagocytic killing capacity of human neutrophils.** (*A)* ATP level of the human neutrophils (PMNs) alone lysed with Triton X-100 subtracted as background from values of samples with both *S. cerevisiae* and PMNs and (*B*) control group of *S. cerevisiae* inoculated at OD_600_ 0.3 and subsequently grown on various Zn conditions (LZM - HZM) as described in Experimental Procedures and Table S2. (*C)* percent killing was determined by treating neutrophils lysed with detergent (2%, Triton X- 100), PMNs infected with opsonized *S. cerevisiae* at a multiplicity of infection (MOI of 2) for 2 h in RPMI 1640 at 37 °C. Viability of *S. cerevisiae* after incubation and PMN lysis was analyzed by quantification of cellular ATP levels using a luciferase-based assay. The results of the independent experiments presented with a number of biological and technical replicates within individual experiments were analyzed as the mean ± SEM using one-way ANOVA with a multiple-comparison test. Additionally, multiple comparisons were made using ANOVA test with corrections from Tukey's, Sidak's, and Dunnett's for all conditions. *p*-values indicate statistical significance at ∗*p* < 0.01.

To investigate how zinc availability for *S. cerevisiae* prior to interaction with PMNs shape the yeast’s susceptibility to neutrophil attack, we precultured *S. cerevisiae* under different conditions ranging from LZM to HZM (0 μM, 0.5 μM, 2.5 μM, and 25 μM). Again, we set to assess yeast viability using cellular ATP levels for quantification. Since live PMNs contain considerable amounts of cellular ATP, we first optimized the lysis step (Fig. 8*A*). Upon detergent-induced lysis of neutrophils, only background levels of ATP signals remained. This treatment spares yeast cells, due to the robustness of fungal cell walls. To ensure equal starting points of the different cultures prior to addition to PMNs, ATP levels of *S. cerevisiae* grown under different zinc-limiting conditions were compared. We observed negligible differences between the cultures of *S. cerevisiae* (Fig. 8*B*). Upon infection of PMNs, we assessed viability of yeast grown under different zinc conditions and calculated the percentage of neutrophil-mediated killing. The percentage of eradicated yeast cells was calculated by normalizing the viability of yeast grown under matching zinc conditions in the absence of PMNs. We found that killing by PMNs is more efficient when *S. cerevisiae* was grown under zinc limiting conditions (Fig. 8*C*). While approximately 80% of *S. cerevisiae* were killed after 2 h of infection, when previously grown under zinc limiting conditions (0 μM, 0.5 μM and 2.5 μM Zn), PMN-mediated killing was reduced to 60% (Fig. 8*C*), when *S. cerevisiae* was previously grown at high zinc concentration (25 μM). A 20% difference in PMN killing might not seem relevant at first glance, however, it is important to notice that this difference solely stems from the zinc content in the growth medium used prior to the killing assay. All other parameters remained the same. Hence, we concluded that PMN killing of microbes increases with lower amounts of zinc available for the microbes. This notion supports our finding that PMNs actively reduce zinc levels of engulfed microbes rather than intoxicating them with high doses of zinc.

## Discussion

Synchrotron nano-XRF and, in particular, hard X-ray nanoprobes beamlines, which are often referred to as ‘nano-imaging facilities’, provide the world’s brightest X-ray beams with 10^11^ photons/s in a focused beam size of 30 to 50 nm (34). As such, nano-XRF allows us to correlate elemental distribution and morphological X-ray imaging of cryogenically frozen (vitrified) cells. Only a few hard X-ray nanoprobes worldwide offer chemical analysis under cryogenic (vitrified) conditions, which circumvents the need for chemical fixation of samples (35). Notably, chemical fixation limits the reliability of elemental analyses. Here, we report the trace element landscape of human PMNs and the model microbe *S. cerevisiae* during phagocytosis. We provide evidence that PMNs effectively deprive zinc from yeast cells during interaction and phagocytosis.

With the appearance of hard X-ray nanoprobes at third- (and fourth) generation synchrotron sources, nanoscopic X-ray imaging and spectroscopic methods under cryogenic conditions offer new possibilities to disentangle metal-related mechanisms of single cells. Cryogenic sample preparation and X-ray data analysis remain cornerstones for obtaining accurate analytical results and correct biological findings. This study is the first to observe dozens of cryogenically frozen PMNs phagocytosing *S. cerevisiae* using the state-of-the-art IDN16A nanoimaging beamline. Hard X-ray-based nanoscopic XRF systems provide a 50-nm spatial resolution, allowing analysis of phagocytosis. Unfortunately, adherence to biosafety restrictions prohibited the use of biosafety level 2 organisms in the high-vacuum beamline setup. A multielement imaging probe of this kind provided us with information on the spatial distribution of several elements, such as phosphorus, sulfur, potassium, and zinc, of vitrified biological samples. XNH revealed our samples' three-dimensional and nanoscopic morphology, confirming the presence of yeast cells within the phagosome. The combination of nano-XRF with XNH uniquely enables element-to-organelle correlation at the nanoscopic level. We were able to identify engulfed *S. cerevisiae*, on the one hand, and lobulated nuclei, phagosomes, and antimicrobial sulfur-rich granules of the PMNs on the other hand.

As PMN phagocytosis relies on the opsonization of microbes (36), we used human serum opsonization of *S. cerevisiae* to increase speed and efficiency for recognition and phagocytosis (37, 38). Our nano-XRF analysis quantitatively compares the elemental content part of the metallome (phosphorus, sulfur, potassium, zinc) of cryo-frozen individual PMNs and opsonized *S. cerevisiae:* (a) in their extracellular environments, (b) in close proximity to PMNs, and (c) inside the PMN phagosomes. Representative nano-XRF images at locations of *S. cerevisiae* phagocytosis clearly showed that the PMN cell membrane started to surround *S. cerevisiae* (Fig. 2). We corroborated internalization of yeast cells by PMNs, utilizing XNH. The XNH imaging confirmed that the analyzed samples captured engulfed *S. cerevisiae* and not yeast cells that were merely attached to PMN surfaces. Neutrophil contact and phagocytosis of *S. cerevisiae* cells led to zinc depletion in yeast cells (Fig. 3, *A* and *B* and Movie S1).

Additionally, we analyzed a sample in which two cells of *S. cerevisiae* are fully phagocytosed, while two other yeast cells are located just outside of the PMN (Fig. 2). As such, this specific XRF map can be seen as a 'nanoscopic snapshot' of the different phases of yeast cells being phagocytosed. The potassium distribution map depicted the general morphology of PMNs and of *S. cerevisiae*. In the sulfur map, we observed sulfur-rich, granular structures that most likely represent neutrophil granules, known to contain high concentrations of antimicrobial proteins (27, 28). As this study focuses on the distribution of zinc during phagocytosis, quantification with statistical analyses was only provided for this element. Described changes for other elements are more descriptive in nature and used to provide a broader context.

Metal ions, particularly zinc, are crucial for the host’s optimal immune function contributing to processes such as phagocytosis, cytokine production, and chemotaxis (39). The immune system’s ability to effectively withhold micronutrients from microorganisms has been termed nutritional immunity (15). Micronutrients are mainly metal ions, such as for instance iron, manganese, and zinc. Nutritional immunity is set in motion by the release of metal ion-binding proteins such as lactoferrin, lipocalin, and S100A8/A9 (15). The cytoplasmic protein S100A8/A9, also referred to as myeloid-related protein (MRP) 8/14 or calprotectin, has been shown to bind copper, manganese, and zinc (40), thereby acting as chelator of essential micronutrients and preventing microbial growth extracellularly (12, 17, 41). It remains to be determined whether S100A8/A9 contributes to deprivation of metal ions also intracellularly, inside immune cells, for instance during phagocytosis.

Interestingly, in our report, areal concentrations of zinc (LOD 0.44 ppm eq. to 6.7 μM) are highest for free *S. cerevisiae*, located outside the PMN, reaching up to 250 ng/cm^2^ (Fig. 4). For phagocytosed *S. cerevisiae*, areal concentrations of zinc are significantly lower, which most likely indicates a deprivation of fungal zinc by the PMN, once the yeast is engulfed. A notable decrease in areal zinc concentration is observed between free and engulfed *S. cerevisiae*, highlighting the role of zinc sequestration in the phagocytic process, as also illustrated in the RGB element map (Fig. 2*E*) with potassium (red), zinc (green), and sulfur (blue) shown. The RGB map provides an additional visual depiction of the element distribution. These findings shed light on the dynamic elemental changes associated with PMN phagocytosis and nutrient sequestration. Macrophages seem to accumulate zinc ions to poison engulfed *Mycobacterium tuberculosis* within the phagolysosome (42). To this date, the mechanisms behind this intoxication remain largely elusive. Based on these findings, speculations emerged that PMNs likely apply similar strategies, a notion stated in research and review articles (15, 43, 44). However, the accumulation of zinc in PMN phagosomes has not been confirmed with image-based approaches. To our knowledge, nano-XRF constitutes the only suitable, label-free methodology with a submicron resolution to quantify the total (*i.e.,* bound and free) zinc concentrations within phagosomes of PMNs. Laser capture microdissection coupled to mass spectrometry as an alternative approach is limited, since the technology most often relies on chemically fixated samples and does not warrant resolution down to the sub-micrometer level (45). Chemical fixation reduces the reliability of trace element quantification since the fixation procedure could introduce leaching or washing out of some trace elements, irrespective of whether those are labile (K, Ca) or bound (Zn, Fe, and Cu) (46). Nanoscale secondary ion mass spectrometry (nano-SIMS) is a powerful alternative. However, nano-SIMS has similar limitations as nano-XRF in regard to sample throughput (47). In addition, nano-SIMS requires biological samples to be compatible with a high-vacuum environment. The surface of the sample needs to be as flat as possible, ideally a few nanometers, which is difficult to achieve for a cellular specimen. In addition, cryogenic sample environments for nano-SIMS are still limited or under development. Our data clearly demonstrates that PMNs, which have engulfed microbes, do not accumulate zinc ions within the phagosome as a general antimicrobial strategy but rather remove zinc from the internalized microbes. As an independent method, we used spinning disc as well as time-lapse fluorescent microscopy of samples with fluorescent zinc probe-labeled *S. cerevisiae* interacting with PMNs. We confirmed that PMNs actively depleted zinc from *S. cerevisiae* during the process of phagocytosis (Fig. 4 and Movie S2). At this point, the exporters involved in the cross-membrane transport of zinc and potential zinc acceptor molecules within the PMN cytoplasm remain to be determined in future studies.

Notably, findings that pathogens rely on proper zinc detoxification capacity for intracellular survival and virulence (42, 43) are not mutually exclusive with regard to our data. Certain pathogens might exceed PMNs’ ability to extract zinc, which ultimately would lead to the accumulation of zinc within the engulfed microbe, requiring detoxification. Given that infiltrating PMNs carry strong zinc-binding proteins, pathogens at the site of infection are probably adapted to a low zinc environment and increase zinc uptake systems before or upon phagocytosis. This scenario, in which both pathogens and immune cells try to snatch zinc, highlights the additional role of PMNs in maintaining an overall immune balance, for which zinc is essential.

During the presented nano-XRF analysis, other metals, such as manganese, iron, and copper, proved to be more difficult to quantify consistently. The main reason for this is that the concentrations of these elements in comparison to zinc are considerably lower within cells and, thus, often close to or below the LOD (Figs. S1–S3 and Table S1). Given the importance of manganese, iron, and copper in immunological processes, their contributions to neutrophil functions will be an interesting topic for potential future studies. In order to improve the LODs of these elements, technical improvements will be needed to reduce the scattering signal from the overall surrounding environment. This could be achieved by implementing improved shielding equipment, for instance. Such improvements contribute to lowering the general background and, thereby, probably will allow for quantification of manganese, iron, and copper.

To investigate whether a drastic increase of zinc concentration could indeed intoxicate a model microorganism, such as *S. cerevisiae*, we performed a series of yeast viability assays under different zinc conditions. After growing yeast in LZM, MZM and HZM, we added a zinc sulfate solution to a final concentration of 500 μM and compared to mock-treated cultures at different time points after the zinc pulse. This concentration exceeds normal zinc levels in human serum 30 to 54 times (31, 32, 33). Using three independent methods, we did not observe an inhibitory or killing effect by the applied zinc pulse, irrespective of the preconditions. Whether *S. cerevisiae* was grown under low- or high-zinc concentrations, the yeast cells remained virtually unaffected by a sudden, drastic increase in the zinc concentration. Notably, *S. cerevisiae* is able to take up more zinc compared to pathogenic microorganisms, such as for instance *Candida albicans* or *C. neoformans*, while the high zinc tolerance is probably attributed to the zinc-dependent alcohol dehydrogenase enzyme Adh1p (48, 49, 50). The element zinc is significantly important for microbes. Approximately 9% of the proteins from the entire yeast proteome are assumed to bind zinc (51), and more than 400 yeast genes play a role under zinc limitation (52), encompassing functions related to zinc homeostasis, endoplasmic reticulum function, oxidative stress resistance, protein folding, vesicular trafficking, and chromatin modification (49, 53, 54).

We aimed to unequivocally determine the strategy related to zinc availability that PMNs use to attack engulfed microbes: Do PMNs extract zinc out of the phagosome with the goal to starve engulfed microbes for zinc, or do PMNs cram the phagosome with excess zinc to intoxicate engulfed microbes? A significant decrease in zinc was observed between free and phagocytosed *S. cerevisiae* within the same and different XRF maps, suggesting an active depletion of zinc by PMNs. In addition, we demonstrated zinc depletion during PMN phagocytosis using both 2D and 3D X-ray nanoimaging as well as time-lapse microscopy, offering a comprehensive view of the process. Interestingly, the neutrophil phagocytic killing capacity of yeast increased in zinc-low environments and decreased in zinc-high environments, again highlighting the importance of this element for microbial anti-phagocytic defense mechanisms. Our findings provide insights into the elemental composition of PMN phagosomes and the consequences on microbial viability under these conditions, with the potential to inspire new targets for improved infection therapies.

## Experimental procedures

### Neutrophil isolation

Fresh 20 ml blood samples were obtained from anonymous healthy donors at the Umeå Blood Central using a Vacutainer K2E 18 mg EDTA (Becton Dickinson, color code: purple). Subsequently, 7 ml of whole blood was layered on 7 ml Histopaque 1119 with a plastic Pasteur pipette in a 15 ml centrifugation tube. Tubes from both donors were centrifuged for 30 min at 800 *g* at room temperature. The serum was discharged, and the peripheral blood monocytes (PBMC) were transferred to another tube and the cells were resuspended using PBS supplement with 0.5% human serum albumin (HSA, Grifols).

A percoll gradient was prepared as previously reported (55). Briefly, 100% isotonic percoll solution was made by mixing 18 ml percoll with 2 ml 10x PBS pH 7.0 (phosphate-buffered saline: 1.4 M NaCl, 27 mM KCl, 90 mM Na_2_HPO_4_, 15 mM KH_2_PO_4_). Then, 85%, 80%, 75%, 70% and 65% of isotonic percoll solutions were prepared by mixing with RPMI-HEPES (Gibco, RPMI without phenol red, substituted with 10 mM HEPES. Then, 2 ml of each percoll concentration was layered in a centrifuge tube, and finally 2 ml of PBMC on top of the gradient.

Gradients were centrifuged for 20 min at 800 g and room temperature. The distinct white layer between clear 70% and 75% percoll in which the neutrophil (PMN) layer is situated was then collected in a new 15-ml centrifugation tube. The cell pellet was resuspended in 1 ml PBS with 0.5% HSA. Cell count provided 1.9 × 10^7^ neutrophils per mL with 92% cell viability and mean neutrophil diameter of 8.8 μm. Cells were diluted in RPMI-HEPES buffer to a concentration of 1 × 10^6^ cells/ml.

### *S. cerevisiae* culture and growth conditions

*S*. *cerevisiae* ('KRY001' diploid strain from ATCC 42800 (strain designation sigma 1278b gpp) was grown in liquid synthetic complete (SC) medium in 10 ml falcon tubes and incubated overnight in a shaking incubator at 30 °C and 180 rpm. All cultures were readjusted to OD_600_ 0.8 to 1.0 before staining or infection experiments. To effectuate phagocytosis, *S. cerevisiae* cultures were opsonized using human pooled serum from 4 donors. For this, cells were spun down and resuspended in PBS with 10% serum for 1.5 h. When complete, *S. cerevisiae* was spun down once, washed with PBS, resuspended in 1 ml, diluted 1:20, and counted.

### Preparation of silicon nitride wafers and μ-slides

Silicon nitride (Si_3_N_4_) membrane frames from Silson (5 × 5 mm^2^ frame size, 1.5 × 1.5 mm^2^ membrane area, 200 μm frame thickness, 500 nm membrane thickness) were coated with 10 nm carbon and glow discharged using a Leica EM ACE. This procedure renders the surface of the wafers more polar, increasing cell adhesion. RPMI medium (200 μl) was loaded in each well of an Ibidi μ-slide. Before insertion of the Si_3_N_4_ frame into the μ-slide well, wafers were rinsed twice in 70% ethanol to sterilize and twice in milliQ water to remove all residual ethanol. Wafers were then placed into the μ-slides using tweezers with their flat surface upwards as this side ensures optimal blotting. We found that the well side of the wafers was prone to capillary effects, potentially resulting in undesirably thicker water layers after blotting. We made sure that no microscopic air bubbles were present between the μ-slide and the wafer membrane, since air bubbles may disturb an even cell distribution.

Except for *S. cerevisiae* from control cultures, 100 μl of PMN solution—containing 1 × 10^5^ PMNs—was added to each μ-slide well and PMNs were incubated (at least) for 1 h at 37 °C. To obtain a 1:1 ratio of PMNs and *S. cerevisiae* in the well, 50 μl of *S. cerevisiae* solution was added, containing 1 × 10^5^ cells. For the control wafers (*i.e.* PMNs alone, *S. cerevisiae* alone), the total volume per well was adjusted with RPMI to 350 μl/well to obtain the same PMN concentration. In total, 3 μ-slides were prepared. One with control cultures of PMNs alone and opsonized *S. cerevisiae* alone. Two other μ-slides contained 1:1 mixture of PMNs with opsonized *S. cerevisiae*. For these two μ-slides, infection times of approximately 2 h and 3 h at the moment of cryofixation were aimed for. Hence, infection with *S. cerevisiae* was performed in a reverse time course, including overhead time for transport and cryofixation. For enabling safe outdoor transport of the cells for cryofixation (vitrification) at the nearby Umeå Centre for Electron Microscopy (UCEM), μ-slides were taped to a T225 culture flask fixed into a Styrofoam box. The culture flask was filled with 37 °C water, enabling it to act as a heat reservoir. At UCEM, each wafer was briefly washed for approx. 5 s *via* slow manual movement through a 0.25 M ammonium formate buffer solution (NH_4_HCO_2_, 2.5455 g dissolved in 160 ml Milli-Q water). This removes the salts medium from the PMN-containing wafers, holding interfering trace level metals. Both sides of the wafer are then automatically blotted with a FEI Vitrobot to remove the excess washing buffer (2s blotting time, 37 °C chamber temperature, blotting force: 0), followed by cryogenic plunge freezing into liquid ethane. After cryofixation, wafers were put in cryogenic vials with magnetic caps from Molecular Dimensions. Cryogenic vials were then inserted in cryocooled ESRF-baskets which were then transported to ESRF using a dry shipper (CX-100, Molecular Dimensions).

### ID16NI beamline ESRF experimental setup

#### General parameters

Scanning nanoscale X-ray fluorescence (XRF) and 3D holography experiments were performed at the ID16A-NI (Nano-Imaging) beamline (UPBL04) at the European Synchrotron Radiation Facility (ESRF). ID16A-NI is a 185 m long beamline capable of hard X-ray microscopy, providing quantitative 3D characterization at nanoscale of both the morphology and the elemental composition of microscopic samples, with main fields of application life science and nanotechnology. At the time of measurements, ID16-NI provided the world's brightest hard X-ray nanofocus, *i.e.*, 2 × 10^11^ photons/s, confined within a beam size of approx. 30 nm horizontally by 30 nm vertically, full-width at half-maximum (FWHM). Incident energy upon the X-ray focusing optics (housed within the high vacuum) sample chamber amounted to 17.1 keV, having a spectral bandwidth ΔE/E of 1% (also 33.6 keV excitation energy can be provided). ID16A beamline techniques used for our study were full-field holography and nanoscale X-ray fluorescence imaging. All measurements were performed under cryogenic conditions. For the latter, the temperature of the holder clamping the sample carrier cube was continuously monitored, remaining constant at approximately −150 °C.

#### Nanoscopic X-ray fluorescence setup

Two pairs of three vertically aligned Silicon Drift Detectors (or SDDs, from Rayspec) were connected to the left flange of the sample chamber (looking downstream), orthogonal to the beam direction. All 6 SDD detectors containing 2048 channels produce an average data output of approximately 80 Mbs^−1^ in scanning mode when using an acquisition time of 50 ms. The detectors have a thin beryllium (Be) window and a 50 mm^2^ active area, each without a collimator. The beryllium window is a compromise between protection of the detector and unnecessary absorption of the XRF signal. In order to have the sample surface perpendicular to the X-ray beam and still be able to collect X-ray fluorescence emerging from the sample, the mean angle between the sample surface and a detector was set to 18°. A positioning stage provides nanoscopic positioning and fast continuous scanning with a maximal speed of 4 μms^−1^. A diode is present behind the sample, determining the flux density for normalization purposes. The X-ray nanobeam can be provided in 'high-dose' (HD) and 'low-dose' (LD) mode, for which the slit opening of the secondary source is set at 50 μm or 10 μm, respectively. More information on the calculation of the limits of detection (LODs) for nano-XRF are provided in the Methods section.

#### 2D/3D in-line X-ray holography

XNH (based on phase contrast imaging) was performed with an ESRF FRELON camera. The field of view camera was 50 μm^2^ upon a CCD array of 2000 × 2000 pixels, resulting in a pixel size of 25 nm. Phase contrast images were taken at 4 different distances within the Fresnel region over a distance of 10 mm behind the focus. For 3D in-line X-ray holography, the sample is rotated over 360° for four different sample-to-detector distances. Holographic reconstruction was performed using ESRF in-house software. The reconstructed phase images then serve as input for the tomographic reconstruction. For visualization of the reconstructed images, ImageJ software was used. Note that a 'missing wedge' problem occurs due to the wafer sample holder, *i.e.*, no phase contrast images can be reconstructed at angles where the frame is parallel to the beam path. Sample drifts throughout rotation (mainly up and downwards) were corrected by fitting a polynomial through specific highlighting spots which stood out in the X-ray phase contrast projections.

### XRF quantification

#### Measurement of standard reference material NIST SRM1577C

For quantification of the neutrophil samples, 21.5 mg of NIST (National Institute of Standards and Technology, U.S. Department of Commerce) standard reference material (SRM)1577C or ‘bovine liver’ was pressed into a self-supporting pellet using a pellet press and evacuable pellet die (Specac Ltd). This resulted in an areal density of the SRM of 16.2 mg/cm^2^). A piece of a few mm^2^ was broken off from this pellet using tweezers and glued with its sides to the supporting frame of a Si_3_N_4_ wafer (without membrane). In this manner, the SRM could be introduced into the ID16NI cryogenic sample environment and measured under similar conditions. Notably, in the online certificate for elemental analysis (56) it is stated that a minimum sample size of 100 mg of the SRM is required. However, a detailed study is provided on the use of synchrotron micro-heterogeneity of metals in low-Z reference materials, where the minimal mass corresponding to an s_material,r_ of 5% was derived (57). In case of NIST SRM1577B, an m_min_,5% = 0.07 μg was obtained. In earlier work, we performed several micro- and nano-XRF heterogeneity studies on the NIST SRM 1577C, such as in (58), depicted in Figure S4 of that publication, for obtaining element maps of the SRM at 50/250 nm step size (58, 59), and a s_heterog_. of 4.5% was derived for zinc determined from 250 independent nano-XRF analyses of the SRM using a step size of 200 nm (60).

First, a ‘coarse’ XRF scan with 250 nm steps was measured at a random location upon the surface of the SRM (60 × 60 points with 100 ms dwell time per point, 15 × 15 μm^2^ area), allowing to verify the homogeneity of the SRM at the microscale. Next, a ‘fine’ XRF scan with 50 nm steps was recorded (100 × 100 points with 50 ms dwell time/point, 5 × 5 μm^2^). The scan was performed at a random position on the SRM, but outside the coarse scan area to avoid sampling areas which may have been affected by sample radiation damage from the previous scan. Notably, experimental conditions of the fine XRF scans were chosen to correspond to those of the analyses of PMNs and *S. cerevisiae* regarding dwell time and pixel size. Although both scans probed a lower mass than suggested (*i.e.* 225 μm^2^ times a total areal mass of 16.2 mg/cm^2^ for the coarse scan results in a probed mass of 0.037 μg), heterogeneity values remained below the 5% level. The considerably high count rate of the SRM measured under high-dose mode for both these measurements result in an undesirable high dead time of the SDD detector. Therefore, a 4.6 μm and 9.2 μm thick gold foil was inserted in the beam path which decreases the flux density with a factor of 3.1 and 9.9 times, respectively (approximately 31 times in total). After insertion of the absorbing gold foils, the mean dead time over all six detectors for the entire map of the SRM decreased to an acceptable 14%. The background radiation of the sample environment was measured by a fine XRF scan with 50 nm steps upon a blank Si_3_N_4_ membrane of 500 nm thickness (40 × 40 points, 50 ms dwell time/point, 2 × 2 μm^2^), this time without the presence of any absorber.

Of note, thin film XRF reference samples have been developed as an alternative to NIST SRM1577C, with homogeneity better than 1% for the full sample area (61). However, these thin film standards have several disadvantages. They contain fewer biologically relevant elements (*i.e.*, iron and copper, but no zinc), allowing only indirect quantification by extrapolation of the elemental yield curve. They break more easily with frequent use, and they are not intended for analysis under cryogenic conditions. Due to these reasons, we did not use thin film XRF reference samples at the time.

#### Normalization and spectral fitting

For XRF quantification, the sum spectrum of the ‘fine’ XRF scan on the SRM (see previous section) was generated by summing all individual point spectra located within the map. Net intensities (I_net_) and background intensities (I_background_) for the elements phosphorus, sulfur, chlorine (Cl), potassium, calcium (Ca), manganese, iron, nickel (Ni), copper, and zinc were determined using AXIL (Advanced X-ray Analysis using Iterative Least squares method, developed at the University of Antwerp) (62). The background modelling algorithm was based on the use of mutually orthogonal polynomials. Both net and background intensities were normalized to dead time, 1 s live time and 200 mA ring current. Normalization of XRF spectra is generally performed by measuring the beam intensity before the sample using sensitive calibrated diodes. Here, the ESRF ring current was preferred to use for normalization of the XRF data for the following reasons: (1) The beam intensity signal I_0_ measured before the sample was inadequate, (2) the beam intensity I_t_ monitored behind the sample varied significantly with the sample ice thickness and also large variations between sample and standard occur, (3) good linearity between ESRF ring current and I_t_ was observed within a single XRF map. Since all samples had an acceptable dead time of maximally 5% at high dose mode, no absorber was required. To calculate the achievable LODs (see next section), net and background intensities of the measured SRM 1577C were corrected to conditions without absorber.

#### Limits of detection (LODs)

Relative limit of detection of an element i was determined by using the following formula:

(Eq. 1) $${LOD}_{relative,i}=\frac{\sqrt{I_{background,i}}\;}{I_{net,i}}\;.\;\omega_{i}.\;\frac{A_{corr,i}\left(SRM\right)}{{\left(\rho d\right)}_{SRM,i}}{.A}_{corr,i}\left(ice\right)$$

whereby I_net,i_ and I_background,i_ denote the net and background intensity of that specific element i determined in the XRF sum spectrum of the SRM using AXIL (see previous section), *ω*_*i*_ the mass fraction of the element as provided in the NIST SRM datasheet (56) and (*ρd*)_*SRM*,*i*_ the areal mass of an element i in the SRM. *A*_*corr*,*i*_(*SRM*) is the (self-)absorption correction factor for the NIST standard measured, expressed in g/cm^2^:

(Eq. 2) $$A_{corr,i}\left(SRM\right)=\frac{1-e^{-\chi_{SRM\left(i\right)}{\;\left(\rho d\right)}_{SRM\left(i\right)}}}{\chi_{SRM\left(i\right)}}$$

In Equation 1, *A*_*corr*,*i*_(*ice*) is the so-called ice correction factor needed when calculating LODs for a (biological) sample which is covered by a uniform ice layer. The absorption correction factor *A*_*corr*,*i*_(*SRM*) for the ice layer covering the sample for a specific element *i* is provided by Equation 3:

(Eq. 3) $$A_{corr,ice}=1/\chi_{ice}\left(i\right)\;{\left(\rho d\right)}_{ice}$$

where parameters *χ*_*ice*_(*i*)*and* and *χ*_*SRM*_(*i*), are factors which correct for absorption effects of exciting and fluorescent X-rays within the ice layer covering the cell and the SRM (more information on both χ parameters is provided in De Samber *et al.*, (2018): *χ*_*ice*_(*i*) is explained in Eq. 16–18, and *χ*_*SRM*(*i*)_ can be calculated based on its constituent elements in NIST certificate and their respective photo-electric X-ray cross sections τ_i_ using Xraylib (63). The respective article also contains information about calculating the ice layer thickness of the sample, provided in Equations (Eq. 2), (Eq. 3), (Eq. 4), (Eq. 5), (Eq. 6), (7), (8), (10), (11).

Note 1: in Equation 1, the variable ‘*A*_*corr*,*i*_(*SRM*)’ is additionally divided by the determined areal mass of the SRM ‘(*ρd*)_*SRM*_’. Since *χ*_*SRM*_ is expressed in *cm*^*2*^*/g* and (*ρd*)_*SRM*_ (is expressed in g/cm^2^, the expression for the relative LOD therefore becomes dimensionless.

Note 2: for calculating *χ*_*SRM*_ in Equation 2, the angle between incoming X-ray beam and sample surface was fixed at 90°. The angle between sample surface and (multi-element) detector was estimated to be 17°.

Note 3: in Equation 3, the areal ice thickness ‘(ρd) _ice_’ is typically expressed in g/cm^2^ and can be obtained by dividing the determined thickness of the ice layer (expressed in μm) by a factor of 1x10^4^, when assuming an ice density of approx. 1 g/cm^3^.

By replacing the weight fraction of element i *ω*_*i*_ by its areal concentration (*ϕd*)_*SRM*(*i*)_ in Equation 1, the areal LOD ‘LOD_areal,i_’ was obtained. By multiplication of LOD_areal,i_ provided in Equation 1 with the beam footprint ‘S_x_’ (estimated to be 30 × 30 nm full-width-at-half-maximum (FWHM), both horizontally and vertically at the time of experiment), the absolute LOD can be obtained as follows:

(Eq. 4) $${LOD}_{absolute,i}={LOD}_{areal,i}.\;S_{X}$$

Alternatively, by multiplication of the weight fraction *ω*_*i*_ (*e.g.* in μg/g) in Equation 1 with the density of the SRM (estimated to be approx. 0.945 g/cm^3^ by determining the thickness of the pellet using a confocal micro-XRF depth scan (64) a volume concentration (μg/cm^3^) is obtained, which can be converted into a molar concentration (or molar LOD, expressed in mole/L) by division with the molar weight M_i_ of element i:

(Eq. 5) $${LOD}_{molar,i}={LOD}_{relative,i}.\rho \text{SRM}\;/\;\text{Mi}$$

Expression of the LOD as molar concentration is useful, due to widespread use of molarity in the field of cell biology. Finally, an atomic LOD (expressed in no. of atoms) can be calculated (representative of the number of atoms that can still be detected in the nanobeam) by dividing the absolute LOD (*e.g.*, expressed in ag or zg) by the molar weight of element i and then multiplying it with the Avogadro’s constant N_A_:

(Eq. 6) $${LOD}_{atomic,i}=\left(\;{LOD}_{absolute,i}/\cdot M_{i}\cdot \right)\;.\;N_{A}$$

Relative, atomic and molar limits of detection (LODs) for typical nano-XRF scanning conditions upon PMNs at ID16NI (*i.e.*, 17 keV excitation, 50 ms dwell time per pixel, high dose mode, no absorbers, normalized to 200 mA ESRF ring current) determined from NIST SRM 1577c (bovine liver) are provided in Figs. S1–S3, expressed as (ppm, μM and number of atoms, respectively). Relative LODs for zinc within a PMN consisting of a 10 μm ice layer (as an acceptable upper limit for ice thickness) were found to be 0.44 *ppm* range, which corresponds to an atomic LOD of 980 zinc atoms per pixel or a molar LOD of 6.7 μM (μM).

### Generation of XRF cluster sum spectra

Summing the individual XRF point spectra belonging to a defined cluster area is common practice for XRF-based quantitative studies, as the generated XRF sum spectrum provides less noisy fluorescence peaks and a background baseline that can be more accurately fitted. Because the XRF counting process follows Poisson-based counting statistics, relative standard deviation of quantitative results derived from the cluster spectra is reduced by a factor of 1/√*t* (see also Equation 10). First, all 2D XRF datasets on PMNs were batch fitted using MICROXRF2, an in-house software written in IDL (Interactive data language, Harris Geospatial Solutions), which calls AXIL in symmetric multi-processing (SMP) mode, finally providing in element maps with raw element intensities. Cluster areas of interest were defined from the element maps *via* manual selection of pixels using the ‘brush’ feature in GIMP (www.gimp.org). For each measurement, the following cluster areas (also referred to as masks) were defined: 1) areal cluster corresponding to the entire PMN using the sulfur elemental map, 2) areal cluster corresponding to *S. cerevisiae* located outside the PMN, 3) areal cluster of *S. cerevisiae* engulfed by the PMN (the latter two both based upon the potassium elemental map) and 4) areal cluster of the background, outside of the PMNs. By subtracting 3 (areal cluster engulfed Sc) from 1 (areal cluster entire PMN containing engulfed Sc.), the area of the PMN reduced with that of the phagocytosed *S. cerevisiae* is obtained.

Similar to the SRM, all PMN and *S. cerevisiae* elemental maps were normalized to 200 mA ESRF ring current, dead time and 1s live time. Using the defined cluster areas, the corresponding cluster XRF sum spectra of 1) entire PMNs, 2) Sc inside and outside PMNs, and 3) engulfed *S. cerevisiae* cluster area removed from entire PMN cluster area can be obtained by summing the individual XRF point spectra belonging to those cluster areas. The Compton signal is representative of the mean electron density of the irradiated area of the sample and therefore the Compton peak may be differing between cluster areas (*e.g.* due to a higher local density), although this effect was minimal in our case since the frozen-hydrated water matrix is the main contributor to the Compton signal. After normalization of the XRF cluster sum spectra to the number of pixels of each cluster area, close overlap of the Compton peaks for each cluster sum spectrum served as a double check for the soundness of the normalization procedure. Hereafter, cluster sum spectra were batch fitted, and finally, element net intensities were saved.

### Estimation of the average ice layer thickness of the entire XRF maps

After blotting and subsequent cryofixation, all samples are covered by a thin layer of vitreous ice of variable dimensions. To account for the ice layer covering the cells, an absorption correction factor is required for accurate XRF quantification. The ice layer thickness was evaluated using the K-K_α_/K_β_ ratio from the entire XRF map sum spectrum, providing an estimate of the average ice thickness above a sample wafer. The estimation of the sample thickness T depends on the determination of the angle θ between sample and the exciting X-ray beam:

(7) $$T\left(\mu m\right)=\log\left[\frac{\frac{K_{\alpha }}{K_{\beta }}/R_{0}}{-\left(\mu_{K\alpha }-\mu_{K\beta }\right)}\right]\;1E4\;\sin\left(\theta \right)$$

For elements with atomic numbers above that of manganese, the influence of the ice layer thickness is negligible for quantification purposes (*i.e.*, below 5%). More information on the use of the K-K_α_/K_β_ method for quantification of XRF cluster sum spectra is provided in a previous publication (58).

### Quantification of nano-XRF maps and XRF cluster spectra

After calculation of the absorption correction factor for ice deposited on the cells, normalized elemental maps providing areal concentrations, for instance as ng/cm^2^, can be quantified using the fundamental parameter (FP) method as referenced previously (65) and according to the following formula:

(8) $$c_{areal,map}=\frac{I\left(x_{map,i}\right)}{Y_{SRM,areal\left(i\right)}}\;\omega_{i}\;A_{corr}\left(ice\right)A_{corr}\left(SRM\right)$$

Here, I(x_map,i_) stands for the dead time-corrected element map of a specific element *i* (also normalized to 1s live time) and Y_SRM_, _areal,i_ for the areal element yield of a specific element i (expressed in counts/(s∗(ng/cm)^2^), determined from the SRM. For explanation of the other variables in Equation 8, we refer to Equation 1. More information on the calculation of the elemental yield curves is provided in (66) (specifically, Equation 6 and Fig. S4 therein).

Besides quantified ‘areal concentration maps’, mean areal concentrations can be obtained by spectral fitting of the normalized cluster sum spectra. The mean areal concentration (*e.g.*, expressed in g/cm^2^) of a particular cluster for an element i is provided by:

(9) careal,cluster(i)=Icluster(i)YSRM,areal(i)ωiAcorr(SRM)Acorr(ice)#pixelscluster(i)

The corresponding relative standard deviation on the value of the areal cluster concentration provided in Equation 9 is governed by Poisson statistics of the cluster sum spectrum:

(10) $$RSD\left(cluster\right)=1/\sqrt{I_{cluster\left(i\right)}*t}$$

where I_cluster(i)_ stands for the total no. of counts for the respective cluster area (*e.g.* nucleus, cytoplasm) for a specific element i (which was initially normalized to 1s for quantification *via* elemental yield and therefore needs to be multiplied by the total measuring time t of that cluster).

When the mean areal concentration of a cluster was determined from the average value of the pixels in the relevant areal concentration map, the corresponding standard deviation would be significantly higher, namely the sum of the relative standard deviations of the individual pixels (also governed by Poisson statistics):

(11) $$RSD\left(cluster\right)=1/\sqrt{\sum \left(I_{pixels}*t\right)}$$

For determining the RSD value of mean areal cluster concentrations provided in Figure 4, *C* and *E*, the first approach (provided in Equation 10) was therefore used.

Moreover, for obtaining the background-corrected areal concentration of the cluster (provided in Fig. 4, *C*–*F*), a subtraction needed to be performed, *i.e.*, the mean areal concentration of the background cluster area was subtracted from the mean areal concentration of the cluster area:

(12) $$\bar{c_{areal,cluster\left(i\right)-bg}}=\bar{c_{areal,cluster\left(i\right)}}-\bar{c_{areal,bg-cluster\;\left(i\right)}}$$

Note 1: To obtain the background-corrected areal concentration maps (provided in Figs. 2 and 3), the mean areal concentration of the background cluster (obtained *via* Equation 12) in those element maps was subtracted from the entire map.

Note 2: To determine the total amount of zinc within a cluster area provided in Figure 4, *D*–*F*, its mean areal concentration was used (*e.g.* in ng/cm^2^ was multiplied by the pixel area and the number of pixels within that cluster).

### Zinc staining of the cell membrane and extracted neutrophils

Neutrophils were extracted from the peripheral blood of healthy human donors as described above. Prior to seeding, cells were counted, and their viability was determined. Subsequently, cells were seeded onto an 8-well slide (μ-Slide 8 Well, ibidi, Cat.No:80826) treated with 10 μg/ml Poly-D-Lysine (Gibco, Cat. No: A3890401) and incubated for 1 h at 37 °C. After incubation, cells were stained with 5 μM FluoZin-3-AM in RPMI 1640 with 10 mM HEPES (both from Gibco, Cat.No:11835030 and Cat.No:15630080) and incubated for additionally 1 h. After incubation, all wells were carefully washed three times with RPMI-1640 before being recovered for 30 min. Remaining RPMI 1640 was removed before staining the PMN cell membranes using 5 μM dioctadecyl-3,3,3′,3′-tetramethylindocarbocyanine perchlorate (DiI; Invitrogen, Cat.No:42364) in RPMI 1640. PMNs were incubated for 15 min before washing 3 times in RPMI-1640. Finally, the PMNs were maintained in RPMI 1640 at 37 °C with 5% CO_2_ prior to subsequent analyses.

### Spinning disk confocal microscopy

After staining the cells, seeded neutrophils were infected with *S. cerevisiae* at a 1:1 ratio unless otherwise stated. Live-cell images were captured every 10 min for 4 h using spinning disk confocal microscopy (Zeiss) controlled by a ZEN software interface (RRID:SCR_013672), built on an Axio Observer Z1 inverted microscope, equipped with a CSU-X1A 5000 spinning disk unit and an EMCCD camera iXon Ultra (ANDOR). The visual fields were chosen manually based on two criteria. Firstly, visual fields of neutrophils and *S. cerevisiae* are in close proximity to each other to increase the chance of interaction. Secondly, all fluorescence channels to detect the different cell types were individually checked to confirm visibility. In total, 6 live-cell imaging sessions were conducted, encompassing 45 visual fields.

### Zinc limitation and zinc pulse assays

To elicit zinc limitation, we used culture medium as previously described with minor modifications (67). The ingredients of LZM are listed in Table S2. Yeast cells were subcultured in SC medium, washed three times with low zinc medium (LZM = 0 μM added ZnSO_4_) or ultrapure water, and inoculated resulting in an optical density at 600 nm (OD_600_) of 0.3 in 5 ml medium in a 50 ml plastic tube. The medium was either LZM, LZM + 2.5 μM ZnSO_4_ (mid-range zinc medium = MZM) or LZM + 25 μM ZnSO_4_ (high zinc medium = HZM). Next, tubes were incubated at 30 °C or 37 °C, 180 rpm for 3 days to reach OD_600_ of 1.5 to 3. Cultures were then seeded into 96-well plates at equal starting concentrations (50,000 cells per well). At the starting point, cell suspensions were treated with addition of 500 μM ZnSO_4_ final concentration (also referred to as ‘zinc pulse’) or mock-treated (mock pulse) with respective medium.

To determine the viability of *S. cerevisiae* we used a luciferase-based assay to quantify cellular ATP. After different time points of incubation in opaque 96-well plates, we added equal volumes of Cell Titer-Glo Reagent (Promega) to pulsed or mock-pulsed *S. cerevisiae* cell suspensions in LZM, MZM, or HZM. Luminescence was measured by a Varioskan flush (Thermo Fisher) microplate reader after 15 to 20 min of tilting incubation at room temperature.

Spot plating technique was also used to determine viability of *S. cerevisiae* upon zinc pulse treatment. Precultured *S. cerevisiae* in LZM, MZM or HZM at 30 °C were seeded into 96-well plates (50,000 cells per well), subjected to a zinc pulse or mock pulse and incubated at 30 °C for up to 4 h. Subsequently, 100 μl cell suspensions of 10-fold serial dilutions were plated on SC agar plates at different time points after treatment. After incubating at 30 °C for 2 to 3 days, CFUs were counted, and the percentage of survival was determined.

Finally, a live and dead staining method was used to determine viability of *S. cerevisiae* upon zinc pulse. Similarly, as described above, *S. cerevisiae* was precultured in LZM, MZM and HZM, seeded into 96-well plates (50.000 cells per well), subjected to a zinc pulse or mock pulse and incubated at 37 °C in a cell imaging multimode reader (Cytation 5, Agilent BioTek). The dyes to stain live and dead yeast cells were added and the assay performed according to the manufacturer’s descriptions (LIVE/DEAD Cell Imaging Kit, Thermo Fisher Scientific). Samples were analyzed at excitation/emission wavelengths of 488 nm/515 nm and 570 nm/602 nm for the green, fluorescent cell stain (live stain) and red fluorescent stain (dead stain), respectively. Corresponding samples lacking the zinc pulse served as 100% controls.

### Quantification of *S. cerevisiae* killing by PMNs using luciferase-based ATP assay

We assessed susceptibility of *S. cerevisiae* to neutrophil attack, when the yeast was grown under different conditions. For this purpose, we infected neutrophils with *S. cerevisiae* at a multiplicity of infection of 2. Prior to infection, *S. cerevisiae* was cultured in LZM, MZM and HZM as described above at 37 °C. PMNs seeded in black clear-bottom 96-well plates to minimize interference for fluorescence assays, were challenged with opsonized *S. cerevisiae* in RPMI 1640 medium incubated at 37 °C and 5% CO_2_ for 2 h. Yeast cell viability was assessed *via* Titer-Glo Reagent similarly as described above. To remove cellular ATP signal from PMNs prior to determination of yeast viability, the immune cells were lysed by detergent (2% Triton X-100) for 30 min. To calculate *S. cerevisiae* viability, the reminiscent ATP levels of lysed neutrophils (negative background control) were subtracted from values of *S. cerevisiae* coincubated with neutrophils as described previously (68, 69). Percentage *S. cerevisiae* killing by PMNs was calculated as [100] − [percentage viability (*S. cerevisiae* + PMNs) of viability (*S. cerevisiae* alone)].

### Statistical analysis

Microsoft Excel was used for basic statistical calculations, while more advanced graphical representations and statistical analyses were performed using GraphPad Prism 8. For statistical analysis ANOVA with Tukey's *post hoc* tests, non-parametric t-tests with Welch's correction, and sample t-tests were performed where appropriate. In addition, one-or two-way ANOVA with Tukey's, Sidak's, or Dunnett's *post hoc* tests were applied for multiple comparisons across all conditions. A *p-*value below 0.05 was considered significant. The degrees of significance are shown as follows: ∗*p* < 0.05, ∗∗*p* < 0.01, ∗∗∗*p* < 0.001, ∗∗∗∗*p* < 0.0001, and “ns” indicates not significant.

## Data availability

All relevant data, including supporting information files, are contained within the manuscript. At the time of measurement, the ESRF Council has recently endorsed the implementation of a data policy for data taken at the ESRF beamlines. The data policy is based on the PaNdata Data Policy which was a deliverable of the European FP7 project PaN-data Europe (http://pan-data.eu/) delivered in 2011. The data policy defines the ESRF as the custodian of raw data and metadata. The metadata is stored in the ICAT metadata catalogue (https://icatproject.org/) which can be accessed online (https://icat.esrf.fr) to browse and download (meta)data. The metadata will be stored in the ICAT metadata catalogue which can be accessed online to browse and download (meta)data. A 3-year embargo period applies after each ESRF measurement during which the experimental team has the right to have sole access to the data, renewable if necessary. The (meta) XRF data related to the experiments of this manuscript: LS-2700 conducted during February 2018 with the accession number doi.org/10.15151/ESRF-ES-85181885 and parts of LS-2768 conducted in June 2018 with the accession number doi.org/10.15151/ESRF-DC-2212115085 are available under a CC-BY-4 license with open access.

## Supporting information

This article contains supporting information. Reference cited include (67, 70).

## Ethics

To isolate neutrophils from peripheral blood, we have acquired venous blood from healthy voluntary donors at the Umeå Blood Central with informed written consent in accordance with the regional ethical board in Umeå. All blood samples were taken by trained personnel and tested negative for HBsAg, HIV 1/2 ab and ag (HIV combo test), anti-HCV, and syphilis. The blood samples were not traceable to the authors and exclusively served as a source for primary neutrophils. No data related to donors was used or saved anywhere, nor was any personal data relevant for the study.

## Conflict of interest

The authors declare that they have no conflicts of interest with the contents of this article.

## References

[1] Gordon S. (2016). Elie metchnikoff, the man and the myth. J. Innate Immun..

[2] Cavaillon J.M. (2011). The historical milestones in the understanding of leukocyte biology initiated by Elie Metchnikoff. J. Leukoc. Biol..

[3] Kaufmann S.H. (2008). Elie Metchnikoff's and Paul Ehrlich's impact on infection biology. Microbes Infect..

[4] Nauseef W.M., Borregaard N. (2014). Neutrophils at work. Nat. Immunol..

[5] Borregaard N. (2010). Neutrophils, from marrow to microbes. Immunity.

[6] Vieira O.V., Botelho R.J., Grinstein S. (2002). Phagosome maturation: aging gracefully. Biochem. J..

[7] Rajarathnam K., Schnoor M., Richardson R.M., Rajagopal S. (2019). How do chemokines navigate neutrophils to the target site: dissecting the structural mechanisms and signaling pathways. Cell Signal..

[8] Rosales C., Uribe-Querol E. (2017). Phagocytosis: a fundamental process in immunity. Biomed. Res. Int..

[9] Sheldon J.R., Skaar E.P. (2019). Metals as phagocyte antimicrobial effectors. Curr. Opin. Immunol..

[10] Fang F.C. (2004). Antimicrobial reactive oxygen and nitrogen species: concepts and controversies. Nat. Rev. Microbiol..

[11] Bairwa G., Hee Jung W., Kronstad J.W. (2017). Iron acquisition in fungal pathogens of humans. Metallomics.

[12] Hood M.I., Skaar E.P. (2012). Nutritional immunity: transition metals at the pathogen-host interface. Nat. Rev. Microbiol..

[13] Chang C.J. (2015). Searching for harmony in transition-metal signaling. Nat. Chem. Biol..

[14] Urban C.F., Ermert D., Schmid M., Abu-Abed U., Goosmann C., Nacken W. (2009). Neutrophil extracellular traps contain calprotectin, a cytosolic protein complex involved in host defense against Candida albicans. PLoS Pathog..

[15] Murdoch C.C., Skaar E.P. (2022). Nutritional immunity: the battle for nutrient metals at the host-pathogen interface. Nat. Rev. Microbiol..

[16] Brophy M.B., Nolan E.M. (2015). Manganese and microbial pathogenesis: sequestration by the Mammalian immune system and utilization by microorganisms. ACS Chem. Biol..

[17] Kehl-Fie T.E., Chitayat S., Hood M.I., Damo S., Restrepo N., Garcia C. (2011). Nutrient metal sequestration by calprotectin inhibits bacterial superoxide defense, enhancing neutrophil killing of Staphylococcus aureus. Cell Host Microbe.

[18] White C., Lee J., Kambe T., Fritsche K., Petris M.J. (2009). A role for the ATP7A copper-transporting ATPase in macrophage bactericidal activity. J. Biol. Chem..

[19] McRae R., Bagchi P., Sumalekshmy S., Fahrni C.J. (2009). In situ imaging of metals in cells and tissues. Chem. Rev..

[20] Adams F. (2014). Spectroscopic imaging: a spatial Odyssey. J. Anal. At. Spectrom..

[21] Perrin L., Roudeau S., Carmona A., Domart F., Petersen J.D., Bohic S. (2017). Zinc and copper effects on stability of tubulin and actin networks in dendrites and spines of hippocampal neurons. ACS Chem. Neurosci..

[22] Chen S., Deng J., Yuan Y., Flachenecker C., Mak R., Hornberger B. (2014). The Bionanoprobe: hard X-ray fluorescence nanoprobe with cryogenic capabilities. J. Synchrotron Radiat..

[23] Wagner D., Maser J., Lai B., Cai Z., Barry C.E., Honer Zu Bentrup K. (2005). Elemental analysis of Mycobacterium avium-, Mycobacterium tuberculosis-, and Mycobacterium smegmatis-containing phagosomes indicates pathogen-induced microenvironments within the host cell's endosomal system. J. Immunol..

[24] Zane Gary M., McCallister G.L. (1996). Quantitative phagocytosis. Am. Biol. Teach..

[25] Rubin-Bejerano I., Fraser I., Grisafi P., Fink G.R. (2003). Phagocytosis by neutrophils induces an amino acid deprivation response in Saccharomyces cerevisiae and Candida albicans. Proc. Natl. Acad. Sci. U. S. A..

[26] Schindler D. (2020). Genetic engineering and synthetic genomics in yeast to understand life and boost biotechnology. Bioengineering (Basel).

[27] Spitznagel J.K. (1990). Antibiotic proteins of human neutrophils. J. Clin. Invest..

[28] Nakatsuji T., Gallo R.L. (2012). Antimicrobial peptides: old molecules with new ideas. J. Invest. Dermatol..

[29] Elmlund D., Le S.N., Elmlund H. (2017). High-resolution cryo-EM: the nuts and bolts. Curr. Opin. Struct. Biol..

[30] Merk A., Bartesaghi A., Banerjee S., Falconieri V., Rao P., Davis M.I. (2016). Breaking cryo-EM resolution barriers to facilitate drug discovery. Cell.

[31] Smith L.D., Garg U., Garg U., Smith L.D. (2017). Biomarkers in Inborn Errors of Metabolism.

[32] Hess S.Y. (2017). National risk of zinc deficiency as estimated by national surveys. Food Nutr. Bull..

[33] Rink L. (2011).

[34] Cesar da Silva J., Pacureanu A., Yang Y., Bohic S., Morawe C., Barrett R. (2017). Efficient concentration of high-energy x-rays for diffraction-limited imaging resolution. Optica.

[35] De Samber B., Meul E., Laforce B., De Paepe B., Smet J., De Bruyne M. (2018). Nanoscopic X-ray fluorescence imaging and quantification of intracellular key-elements in cryofrozen Friedreich's ataxia fibroblasts. Plos One.

[36] Dunkelberger J.R., Song W.C. (2010). Complement and its role in innate and adaptive immune responses. Cell Res..

[37] Parker L.C., Whyte M.K.B., Dower S.K., Sabroe I. (2005). The expression and roles of Toll-like receptors in the biology of the human neutrophil. J. Leukoc. Biol..

[38] Arnaout M.A. (1990). Structure and function of the leukocyte adhesion molecules CD11 CD18. Blood.

[39] Prasad A.S. (2008). Zinc in human health: effect of zinc on immune cells. Mol. Med..

[40] Gilston B.A., Skaar E.P., Chazin W.J. (2016). Binding of transition metals to S100 proteins. Sci. China Life Sci..

[41] Wątły J., Potocki S., Rowińska-Żyrek M. (2016). Zinc homeostasis at the bacteria/host interface—from coordination chemistry to nutritional immunity. Chemistry–A Eur. J..

[42] Botella H., Peyron P., Levillain F., Poincloux R., Poquet Y., Brandli I. (2011). Mycobacterial p(1)-type ATPases mediate resistance to zinc poisoning in human macrophages. Cell Host Microbe.

[43] Ong C.L., Gillen C.M., Barnett T.C., Walker M.J., McEwan A.G. (2014). An antimicrobial role for zinc in innate immune defense against group A streptococcus. J. Infect. Dis..

[44] Djoko K.Y., Ong C.L., Walker M.J., McEwan A.G. (2015). The role of copper and zinc toxicity in innate immune defense against bacterial pathogens. J. Biol. Chem..

[45] Datta S., Malhotra L., Dickerson R., Chaffee S., Sen C.K., Roy S. (2015). Laser capture microdissection: big data from small samples. Histol. Histopathol.

[46] Pushie M.J., Pickering I.J., Korbas M., Hackett M.J., George G.N. (2014). Elemental and chemically specific X-ray fluorescence imaging of biological systems. Chem. Rev..

[47] De Samber B., De Rycke R., De Bruyne M., Kienhuis M., Sandblad L., Bohic S. (2020). Effect of sample preparation techniques upon single cell chemical imaging: a practical comparison between synchrotron radiation based X-ray fluorescence (SR-XRF) and Nanoscopic Secondary Ion Mass Spectrometry (nano-SIMS). Anal. Chim. Acta.

[48] Raj S.B., Ramaswamy S., Plapp B.V. (2014). Yeast alcohol dehydrogenase structure and catalysis. Biochemistry.

[49] Wehmeier S., Morrison E., Plato A., Raab A., Feldmann J., Bedekovic T. (2020). Multi trace element profiling in pathogenic and non-pathogenic fungi. Fungal Biol..

[50] Eide D.J. (2006). Zinc transporters and the cellular trafficking of zinc. Biochim. Biophys. Acta (BBA).

[51] Andreini C., Banci L., Bertini I., Rosato A. (2006). Zinc through the three domains of life. J. Proteome Res..

[52] North M., Steffen J., Loguinov A.V., Zimmerman G.R., Vulpe C.D., Eide D.J. (2012). Genome-wide functional profiling identifies genes and processes important for zinc-limited growth of Saccharomyces cerevisiae. Plos Genet..

[53] Fleming S.B. (2016). Viral inhibition of the IFN-induced JAK/STAT signalling pathway: development of live attenuated vaccines by mutation of viral-encoded IFN-antagonists. Vaccines.

[54] Huang J., Canadien V., Lam G.Y., Steinberg B.E., Dinauer M.C., Magalhaes M.A. (2009). Activation of antibacterial autophagy by NADPH oxidases. Proc. Natl. Acad. Sci. U. S. A..

[55] Ermert D., Zychlinsky A., Urban C. (2009). Fungal and bacterial killing by neutrophils. Methods Mol. Biol..

[56] (NIST), N. I. o. S. a. T. (2025).

[57] Kempenaers L., Vincze L., Janssens K. (2000). The use of synchrotron micro-XRF for characterization of the micro-heterogeneity of heavy metals in low-Z reference materials. Spectrochimica Acta B.

[58] De Samber B., Meul E., Laforce B., De Paepe B., Smet J., De Bruyne M. (2018). Nanoscopic X-ray fluorescence imaging and quantification of intracellular key-elements in cryofrozen Friedreich’s ataxia fibroblasts. PLoS One.

[59] De Samber B., Silversmit G., De Shcamphelaere K., Evens R., Schoonjans T., Vekemers B. (2010). J. Anal. Spectrom..

[60] De Samber B. (2010).

[61] Dresden A. (2022).

[62] Vekemans B., Janssens K., Vincze L., Adams F., Vanespen P. (1994). Analysis of X-RAY-SPECTRA by iterative least-squares (AXIL) - new developments. X-ray Spectrom..

[63] Schoonjans T., Brunetti A., Golosio B., Sanchez del Rio M., Solé V.A., Ferrero C. (2011). The xraylib library for X-ray–matter interactions. Recent developments. Spectrochimica Acta Part B.

[64] De Samber B., Silversmit G., De Schamphelaere K., Evens R., Schoonjans T., Vekemans B. (2010). Element-to-tissue correlation in biological samples determined by three-dimensional X-ray imaging methods. J. Anal. At. Spectrom..

[65] Szalóki I., Gerényi A., Radócz G., Lovas A., De Samber B., Vincze L. (2017). FPM model calculation for micro X-ray fluorescence confocal imaging using synchrotron radiation. J. Anal. At. Spectrom..

[66] De Samber B., Niemiec M.J., Laforce B., Garrevoet J., Vergucht E., De Rycke R. (2016). Probing intracellular element concentration changes during neutrophil extracellular trap formation using synchrotron radiation based X-ray fluorescence. PLoS One.

[67] Crawford A.C., Lehtovirta-Morley L.E., Alamir O., Niemiec M.J., Alawfi B., Alsarraf M. (2018). Biphasic zinc compartmentalisation in a human fungal pathogen. Plos Pathog..

[68] Shankar M., Lo T.L., Traven A. (2020). Natural variation in clinical isolates of Candida albicans modulates neutrophil responses. mSphere.

[69] Unger L., Skoluda S., Backman E., Amulic B., Ponce-Garcia F.M., Etiaba C.N. (2023). *Candida albicans* induces neutrophil extracellular traps and leucotoxic hypercitrullination via candidalysin. EMBO Rep..

[70] Crawford A.M., Deb A., Penner-Hahn J.E. (2019). M-BLANK: a program for the fitting of X-ray fluorescence spectra. Synchrotron Radiat..

