# Supplementary material for: Nanoscale chemical imaging of phagocytosis: A battle for metals between host and microbe
Source: J Biol Chem. 2025 Jul 16;301(9):110485. doi: 10.1016/j.jbc.2025.110485 (PMC12405629; doi:10.1016/j.jbc.2025.110485)
Supplement: Supporting Information [file mmc1.pdf]

Nanoscale Chemical Imaging of Phagocytosis: A Battle for Metals between Host and Microbe

Nadeem Ullah[1,2], Björn De Samber[3#], Nathalie Uwamahoro[1,2], Stijn J.M Van Malderen[3], Linda Sandblad[4,5], Sylvain Bohic[6], Peter Cloetens[6], Laszlo Vincze[3], Constantin F. Urban[1,2] *

[1] Department of Clinical Microbiology, Umeå University, Umeå, Sweden

[2]Umeå Centre for Microbial Research (UCMR), Umeå University, Umeå, Sweden

[3]Department of Analytical Chemistry, Ghent University, Ghent, Belgium

[4]Department of Chemistry, Umeå University, Umeå, Sweden

[5]Umeå Centre for Electron Microscopy, Umeå University, Umeå, Sweden

[6]European Synchrotron Radiation Facility, Grenoble, France

[#]Current address: imec Vision Lab, Department of Physics, University of Antwerp, Antwep, Belgium

*Corresponding author: constantin.urban@umu.se

Running title

Nanoscale Imaging of Phagocytosis: Nutritional Immunity

The Supporting Information file includes

- Supporting Figure S1-7
- Supporting Table S1-2
- Caption for Supporting Movies S1-2

Supporting Figures

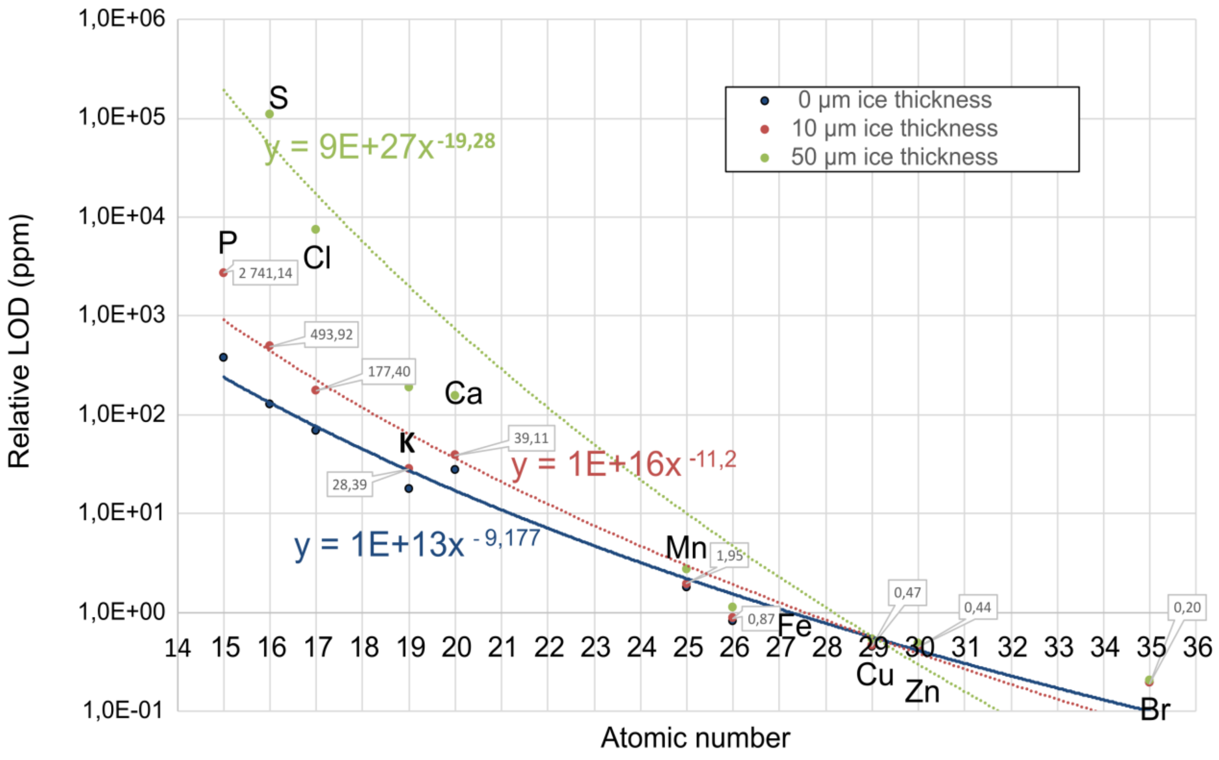

Figure S1: Relative limits of detection (LODs, expressed in parts-per-million or ppm) at ID16NI for typical nano-XRF scanning conditions (17 keV excitation energy, 50 ms dwell time/pixel, high dose mode, no absorbers, normalized to 200 mA ESRF ring current) for NIST SRM 1577c (bovine liver). LODs are calculated for a virtual ice layer thickness of 0 µm (blue curve), 10 µm (red curve) and 50 µm (green curve). Exact values are indicated with a data-lay-out. An exponential curve has been fitted for each ice layer thickness.

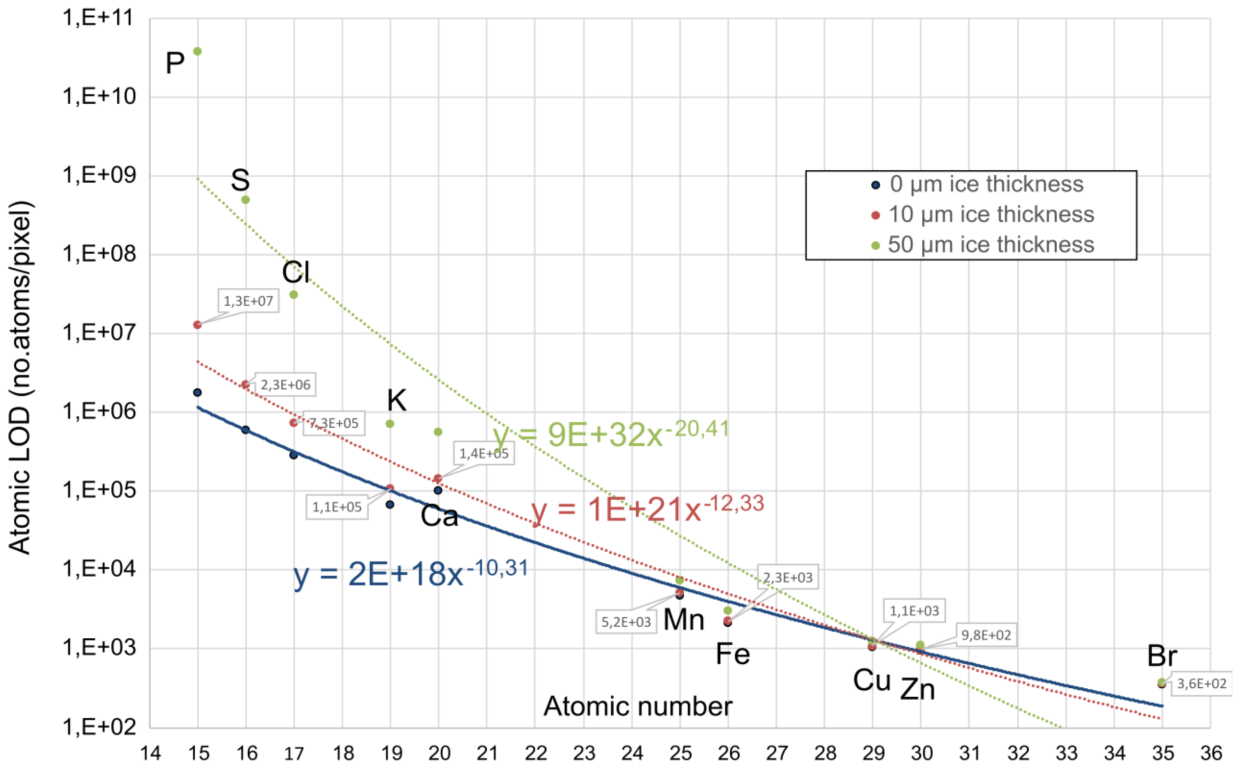

Figure S2: Atomic LODs (in no. of atoms/pixel) at ID16NI for typical nano-XRF scanning conditions (17 keV excitation energy, 50 ms dwell time/pixel, high dose mode, no absorbers, normalized to 200 mA ESRF ring current) for NIST SRM 1577c (bovine liver). LODs are calculated for a virtual ice layer thickness of 0 µm (blue curve), 10 µm (red curve) and 50 µm (green curve). Exact values are indicated with a data-lay-out. An exponential curve has been fitted for ice layer thickness.

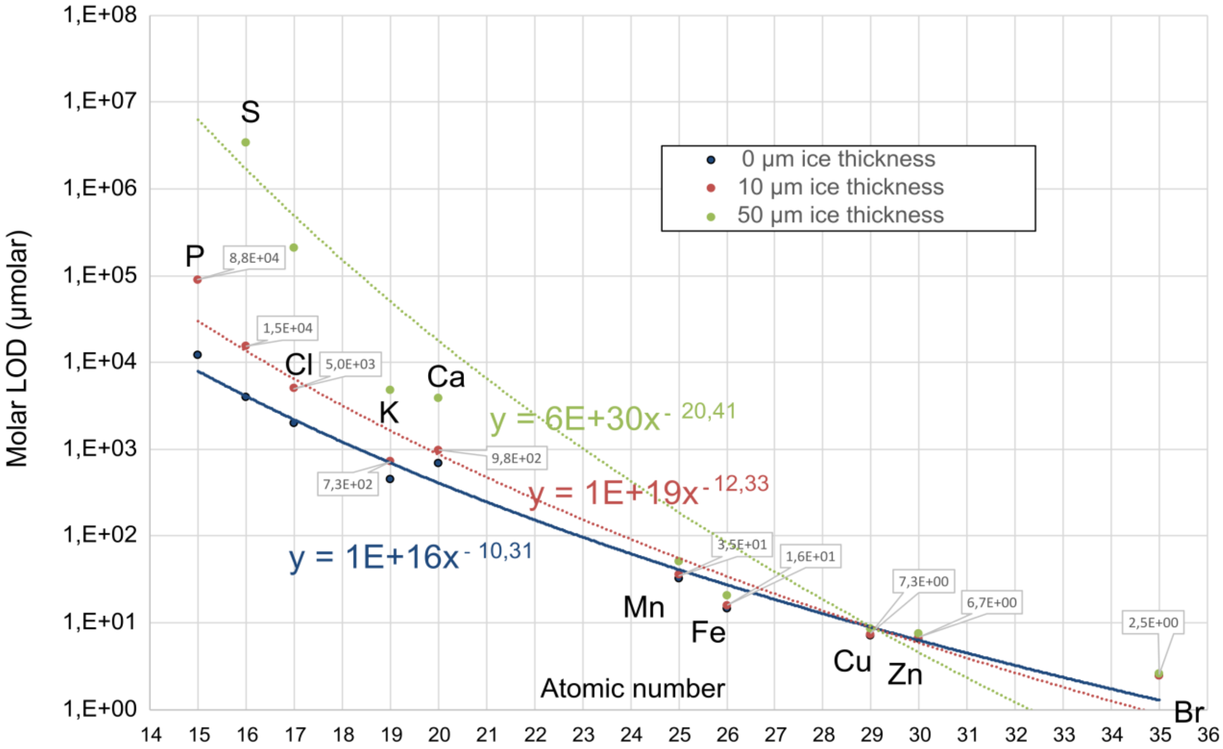

Figure S3: Molar LODs (in µM) at ID16NI for typical nano-XRF scanning conditions (17 keV excitation energy, 50 ms dwell time/pixel, high dose mode, no absorbers, normalized to 200 mA ESRF ring current) for NIST SRM 1577c (bovine liver). LODs are calculated for a virtual ice layer thickness of 0 µm (blue curve), 10 µm (red curve) and 50 µm (green curve). Exact values are indicated with a data-lay-out. An exponential curve has been fitted for each ice layer thickness.

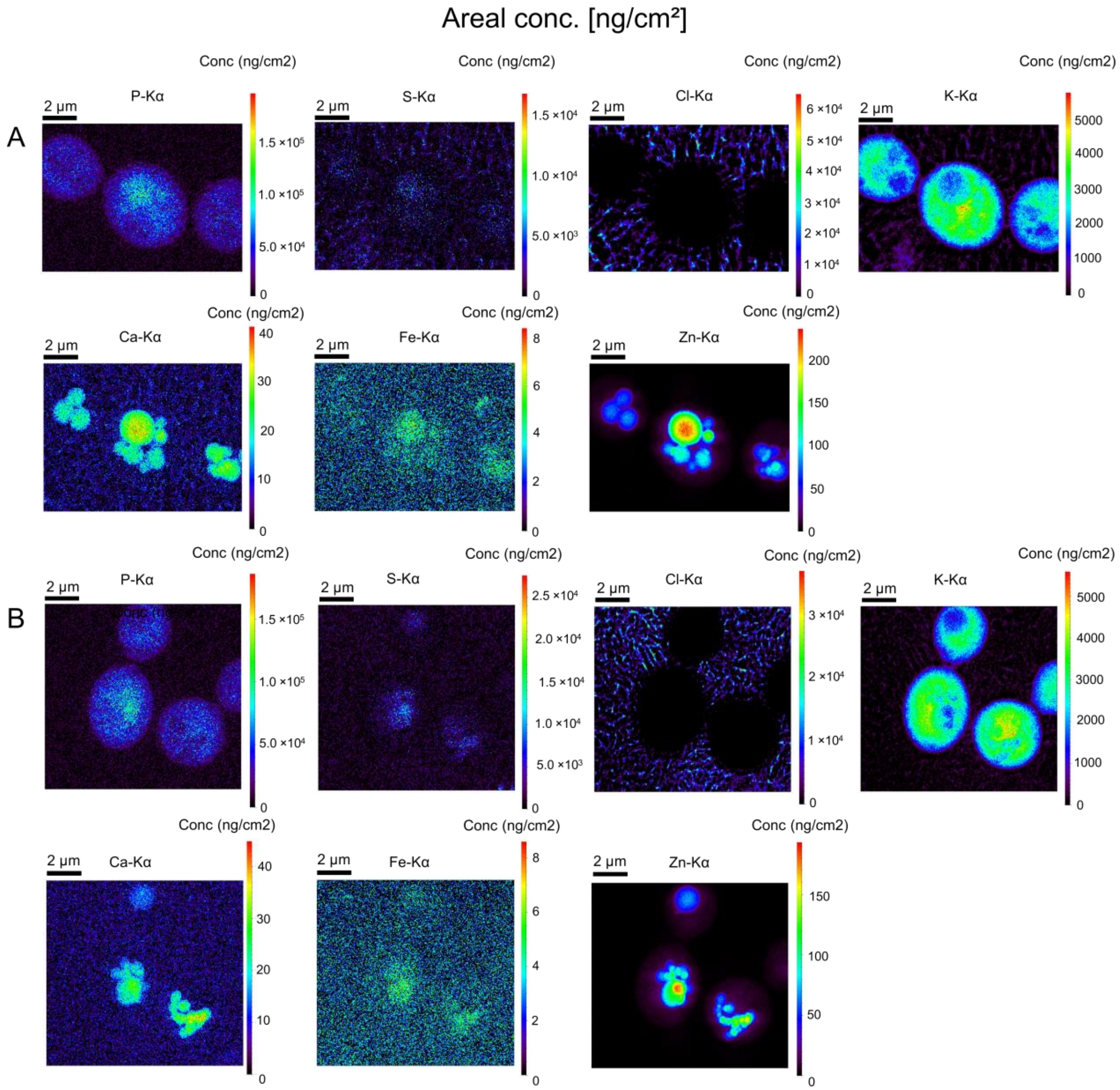

Figure S4: Normalized elemental concentration heat maps of representative yeast cultured in control conditions. (**A-B)** Quantified elements shown are P, S, Cl, K, Ca, Fe, and Zn. For each elemental map, the mean background value was subtracted. Pixel size is 50 nm, dwell time was 50 ms. Quantification was based upon the measurement of a thin flake of NIST SRM1577C (bovine liver). In the potassium map, the oval shapes of the yeast cells are indicated. Areal concentrations were calculated and expressed in ng/cm^2, equivalent to ppm (parts-per-million) when the cell is assumed to be contained within a 10 micron water layer. All elemental maps were normalized to 200 mA ESRF ring current and corrected for dead time. Absorption due to the ice layer covering the cell was corrected for using fundamental parameters. Note that the signal from the element Cl

is somewhat ‘bleeding’ into the S map (i.e. some Cl features and patterns seem to be present in the S-map due to inaccuracies in background estimation but are in fact artificial) (70). The high-quality images with the beamline microscope were obtained by a 2µm spatial resolution detector as demonstrated by the 2µm scale bar.

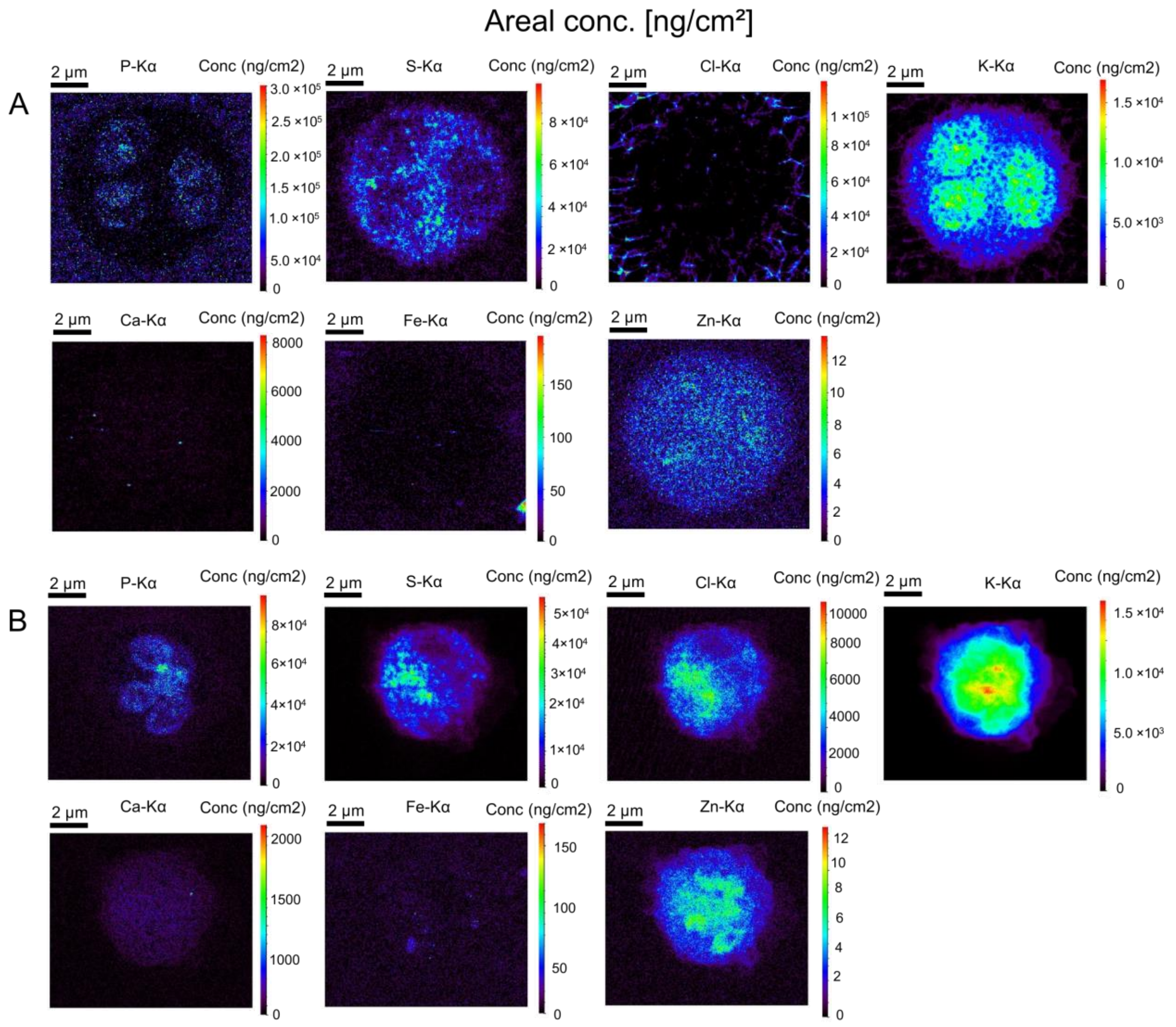

Figure S5: Shown are normalized elemental concentration heat maps of a representative, unstimulated human PMN cultured in control conditions. Freshly isolated PMNs were seeded on a Si3N4 membrane and subsequently freeze-dried. (**A-B)** Quantified elements are P, S, Cl, K, Ca, Fe, and Zn. For each elemental map, the mean background value was subtracted. Pixel size is 50 nm, dwell time was 50 ms. Quantification was based upon the measurement of a thin flake of NIST SRM1577C (bovine liver). In the potassium map, the spherical structure of the PMN is

indicated. The uninfected PMN shows some deformations at the surface of the cell. Areal concentrations were calculated and expressed in ng/cm^2, equivalent to ppm (parts-per-million) when the cell is assumed to be contained within a 10 microns water layer. All elemental maps were normalized to 200 mA ESRF ring current and corrected for dead time, absorption due to the ice layer covering the cell was corrected for using fundamental parameters. The high-quality images with the beamline microscope were obtained by a 2µm spatial resolution detector as demonstrated by the 2µm scale bar.

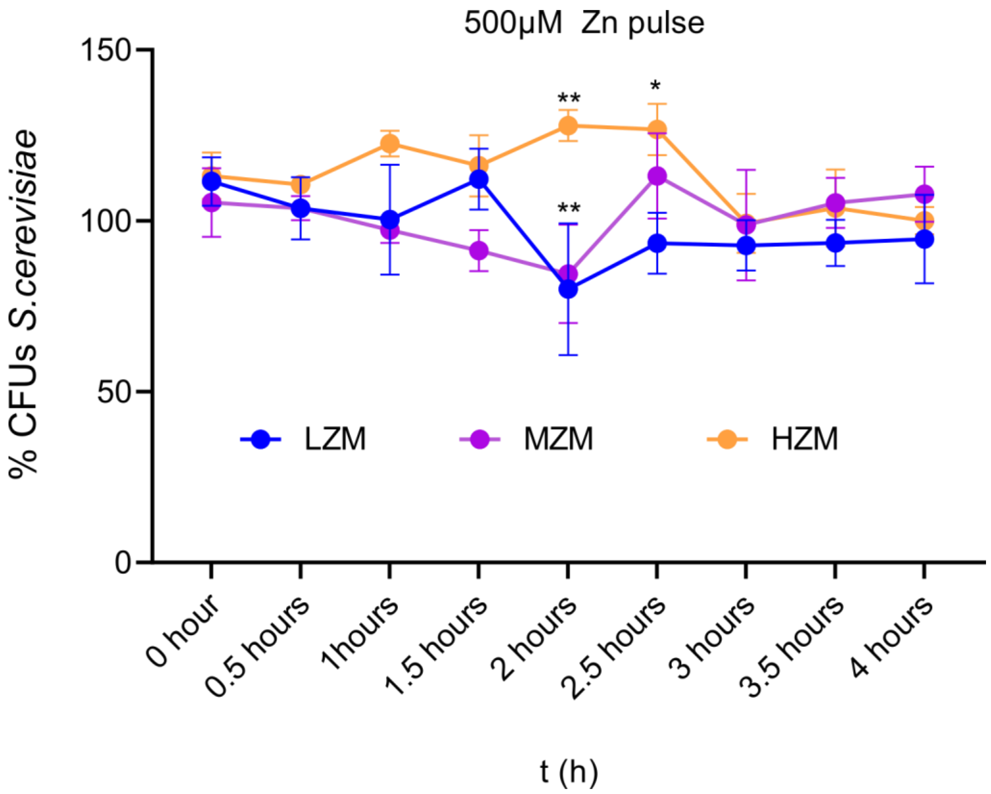

Figure S6: **Survival assay of *S. cerevisiae* following Zn pulse.** Percentage (%) of the *S. cerevisiae* CFUs following 500µM Zn pulse. The results were shown as the mean ± SEM of three independent experiments (n=3) where n represents the number of biological replicates, with multiple technical replicates within individual experiments. *P*-value were calculated using a one-way ANOVA with post-hoc Tukey's test adjustment for multiple comparisons, $**p < 0.001$, $*p < 0.05$ indicate statistical significance.

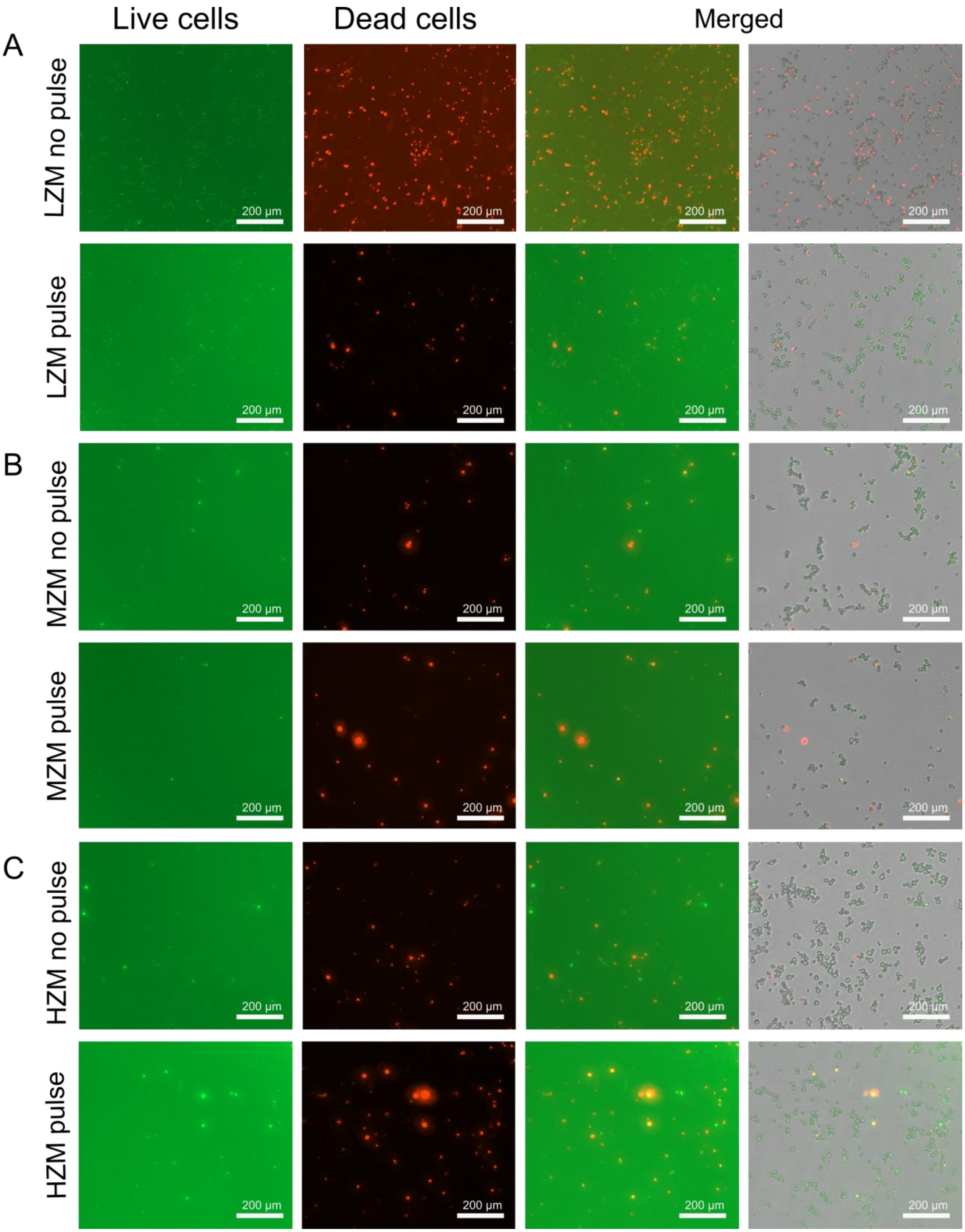

Live cells
Dead cells
Merged
A
B
C
LZM no pulse
LZM pulse
MZM no pulse
MZM pulse
HZM no pulse
HZM pulse
200 µm

Figure S7: **Microscopic representation of *S. cerevisiae* following exposure to Zn pulse.** The representative microscopic images at 20X magnification recorded using Cytation 5 cell imaging reader (BioTek) show *S. cerevisiae* cells 2.5 h after exposure to a Zn pulse (500 µM) at 37 °C. (**A)** Cells were used for the assay after they have been cultured in a low Zn (LZM), (**B)** a medium Zn (MZM) or (**C)** a high Zn environment (HZM). Notably, visualization of live cell staining with green fluorescence (ex/em 488 nm/515 nm) was challenging due to the high background level compared to red fluorescence (ex/em 570 nm/602 nm) and low background levels. In the last column of images, a differential interference contrast (DIC) image was additionally added to the merged fluorescence. Scale bar represents 200 µm.

Supporting Tables

Table S1. Limited of detection

At. No.+AA2:I36Element	YIELD		LOD				
Ice thickness	areal_yield	absolute	relative	areal	abs.	atomic	molar
T= 0 µm ice	(cts/s/ng/cm²)	(cts/ag)	(ppm)	(ng/cm²)	(ag)	(no. atoms)	(µM)
15 P	5,1E-03	3,4E-01	3,7E+02	6,0E+03	9,1E+01	1,8E+06	1,2E+04
16 S	1,5E-02	9,8E-01	1,3E+02	2,1E+03	3,1E+01	5,9E+05	4,0E+03
17 Cl	2,8E-02	1,8E+00	7,0E+01	1,1E+03	1,7E+01	2,9E+05	2,0E+03
19 K	9,6E-02	6,4E+00	1,8E+01	2,9E+02	4,3E+00	6,6E+04	4,5E+02
20 Ca	5,7E-02	3,8E+00	2,8E+01	4,5E+02	6,7E+00	1,0E+05	6,9E+02
25 Mn	9,7E-01	6,5E+01	1,8E+00	2,9E+01	4,3E-01	4,8E+03	3,2E+01
26 Fe	2,4E+00	1,6E+02	8,1E-01	1,3E+01	2,0E-01	2,1E+03	1,5E+01
29 Cu	4,7E+00	3,1E+02	4,5E-01	7,3E+00	1,1E-01	1,0E+03	7,1E+00
30 Zn	4,8E+00	3,2E+02	4,3E-01	6,9E+00	1,0E-01	9,6E+02	6,5E+00
35 Br	9,0E+00	6,0E+02	1,9E-01	3,1E+00	4,7E-02	3,6E+02	2,4E+00
T= 10 µm ice							
15 P	6,9E-04	4,6E-02	2,7E+03	4,4E+04	6,7E+02	1,3E+07	8,8E+04
16 S	3,8E-03	2,6E-01	4,9E+02	8,0E+03	1,2E+02	2,3E+06	1,5E+04
17 Cl	1,1E-02	7,2E-01	1,8E+02	2,9E+03	4,3E+01	7,3E+05	5,0E+03
19 K	6,0E-02	4,0E+00	2,8E+01	4,6E+02	6,9E+00	1,1E+05	7,3E+02
20 Ca	4,0E-02	2,7E+00	3,9E+01	6,3E+02	9,5E+00	1,4E+05	9,8E+02
25 Mn	8,9E-01	6,0E+01	1,9E+00	3,2E+01	4,7E-01	5,2E+03	3,5E+01
26 Fe	2,2E+00	1,5E+02	8,7E-01	1,4E+01	2,1E-01	2,3E+03	1,6E+01
29 Cu	4,6E+00	3,0E+02	4,7E-01	7,5E+00	1,1E-01	1,1E+03	7,3E+00
30 Zn	4,6E+00	3,1E+02	4,4E-01	7,1E+00	1,1E-01	9,8E+02	6,7E+00
35 Br	8,9E+00	5,9E+02	2,0E-01	3,2E+00	4,8E-02	3,6E+02	2,5E+00
T= 50 µm ice							
15 P	2,4E-07	1,6E-05	8,0E+06	1,3E+08	1,9E+06	3,8E+10	2,6E+08
16 S	1,7E-05	1,2E-03	1,1E+05	1,8E+06	2,6E+04	5,0E+08	3,4E+06
17 Cl	2,6E-04	1,7E-02	7,4E+03	1,2E+05	1,8E+03	3,1E+07	2,1E+05
19 K	9,0E-03	6,0E-01	1,9E+02	3,1E+03	4,6E+01	7,1E+05	4,8E+03
20 Ca	1,0E-02	6,7E-01	1,6E+02	2,5E+03	3,8E+01	5,7E+05	3,9E+03
25 Mn	6,3E-01	4,2E+01	2,7E+00	4,4E+01	6,7E-01	7,3E+03	5,0E+01
26 Fe	1,7E+00	1,1E+02	1,1E+00	1,8E+01	2,8E-01	3,0E+03	2,0E+01
29 Cu	4,0E+00	2,6E+02	5,4E-01	8,7E+00	1,3E-01	1,2E+03	8,4E+00
30 Zn	4,1E+00	2,8E+02	4,9E-01	8,0E+00	1,2E-01	1,1E+03	7,5E+00
35 Br	8,5E+00	5,6E+02	2,1E-01	3,3E+00	5,0E-02	3,8E+02	2,6E+00

Table S1: Limits of detection (LODs) at ID16NI for typical nano-XRF scanning conditions (17 keV excitation energy, 50 ms dwell time/pixel, high dose mode, no absorbers, normalized to 200 mA ESRF ring current) for NIST SRM 1577c (bovine liver). Since the LOD for an element is dependent on the ice layer covering the sample, which is of relevance in particular for the lower atomic number elements, LODs are calculated for a virtual ice layer thickness of 0 µm (blue curve), 10 µm (red curve) and 50 µm (green curve).

Table S2. Limited Zinc Media Preparation (LZM)

Stock	Fold conc.	Component	Stock conc. (M)	Final conc. (M)	Vol.
1	500	$Na_2EDTA.2H_2O$	$5.0x10^{-1}$	$1.0x10^{-3}$	1ml
2	100	$MgSO_4.7H_2O$<br>NaCl	$5.0x10^{-1}$<br>$1.0x10^{-1}$	$5.0x10^{-3}$<br>$1.0x10^{-3}$	5ml
3	100	$CaCl_2.2H_2O$	$1.0x10^{-1}$	$1.0x10^{-3}$	5ml
4	100	uridine<br>L-histidine<br>L-leucine<br>L-lysine	$4.0x10^{-2}$<br>$5.0x10^{-2}$<br>$7.6x10^{-2}$<br>$7.0x10^{-2}$	$4.0x10^{-4}$<br>$5.0x10^{-4}$<br>$7.6x10^{-4}$<br>$7.0x10^{-4}$	5ml
5	100	$(NH_4)_2SO_4$	3.8	$3.8x10^{-2}$	5ml
6	100	KH_2PO_4	$1.0x10^{-1}$	$1.0x10^{-3}$	5ml
7	50	$Na_3citrate.2H_2O$	1.0	$2.0x10^{-2}$	10ml
8	20	D-glucose	$2.2x10^{-1}$	$1.1x10^{-2}$	25ml
9	1000	d-biotin<br>Ca pantothenate<br>myo-inositol<br>pyridoxin<br>thiamine. HCl	$1.6x10^{-5}$<br>$1.7x10^{-3}$<br>$1.0x10^{-2}$<br>$2.0x10^{-3}$<br>$1.0x10^{-3}$	$1.6x10^{-8}$<br>$1.7x10^{-6}$<br>$1.0x10^{-5}$<br>$2.0x10^{-6}$<br>$1.0x10^{-6}$	0.5ml
10	10000	H_3BO_3<br>KI<br>$Na_2MoO_4.2H_2O$	$1.0x10^{-1}$<br>$5.0x10^{-3}$<br>$1.0x10^{-2}$	$1.0x10^{-5}$<br>$5.0x10^{-7}$<br>$1.0x10^{-6}$	50µl

Table S2: To prepare the LZM media, the stock solutions were sequentially added to ultra-pure water, filter-sterilized, and stored in 50 ml sterile plastic tubes. To generate the respective metal limited media (LZM, LZM+0.5µM Zn, LZM+2.5µM Zn & LZM+25µM Zn), the following transition metals were added: FeCl (6.17 µM), $MnSO_4$ (13.24 µM), $CuSO_4$ (0.3 µM) and $ZnSO_4$ (25 µM). To generate LZM pH 7.3, media was alkalinized with NaOH and buffered with 50 mM HEPES pH 7 (67).

Caption for Supporting Movie S1:

Time-lapse of a 3D in-line X-ray holography. The entire sequence of reconstructed slices was added and rendered into a time-lapse video to illustrate that samples investigated at ID16NI were PMNs with phagocytosed yeast inside, not on top or below the phagocytic cell.

Caption for Supporting Movie S2:

Entire time-lapse video of stills shown in Figure 5A.
